# Extrachromosomal DNA amplicons in antimalarial‐resistant *Plasmodium falciparum*

**DOI:** 10.1111/mmi.14624

**Published:** 2020-11-19

**Authors:** Jennifer M. McDaniels, Adam C. Huckaby, Sabrina A. Carter, Sabrina Lingeman, Audrey Francis, Molly Congdon, Webster Santos, Pradipsinh K. Rathod, Jennifer L. Guler

**Affiliations:** ^1^ Department of Biology University of Virginia Charlottesville VA USA; ^2^ Department of Chemistry Virginia Tech Blacksburg VA USA; ^3^ Departments of Chemistry and Global Health University of Washington Seattle WA USA; ^4^ Division of Infectious Diseases and International Health Department of Medicine University of Virginia Charlottesville VA USA

**Keywords:** adaptation, extrachromosomal DNA, genome, malaria, *Plasmodium*, resistance

## Abstract

Extrachromosomal (ec) DNAs are genetic elements that exist separately from the genome. Since ecDNA can carry beneficial genes, they are a powerful adaptive mechanism in cancers and many pathogens. For the first time, we report ecDNA contributing to antimalarial resistance in *Plasmodium falciparum,* the most virulent human malaria parasite. Using pulse field gel electrophoresis combined with PCR‐based copy number analysis, we detected two ecDNA elements that differ in migration and structure. Entrapment in the electrophoresis well and low susceptibility to exonucleases revealed that the biologically relevant ecDNA element is large and complex in structure. Using deep sequencing, we show that ecDNA originates from the chromosome and expansion of an ecDNA‐specific sequence may improve its segregation or expression. We speculate that ecDNA is maintained using established mechanisms due to shared characteristics with the mitochondrial genome. Implications of ecDNA discovery in this organism are wide‐reaching due to the potential for new strategies to target resistance development.

## Graphical abstract

The most lethal malaria parasite, *Plasmodium falciparum,* rapidly adapts to acquire drug resistance. For the first time, we report that highly resistant *Plasmodium* parasites carry additional copies of resistance‐conferring genes separate from the genome, which are known as extra‐chromosomal DNA (ecDNA). This finding provides a new model to study a fundamental mechanism of adaptation that is relevant in many microbes and may lead to a new way to target malaria.

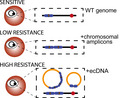

## 1 INTRODUCTION

A major factor that contributes to genome plasticity in a variety of organisms is extrachromosomal (ec) DNA. EcDNA contains extra gene copies that exist outside of the genome and are often observed at high copy numbers in organisms under strong selection. As evidence for their contribution to genome plasticity, genes that confer fitness benefits are enriched in ecDNA (Albertson, 2006; Beverley, 1988; Dillon et al., 2015; Genois et al., 2014; Hastings et al., 2009; Leprohon et al., 2014; McGill et al., 1993; Møller et al., 2015; Shibata et al., 2012; Verhaak et al., 2019; Wagner and So, 1992). Although first discovered in the *Leishmania* protozoan parasite (Beverley et al., 1984), ecDNA has now been reported in various eukaryotes including yeast (Møller et al., 2015), human cancers (Albertson, 2006; Hastings et al., 2009; McGill et al., 1993; Verhaak et al., 2019; Wu et al., 2019), mammalian cells (Dillon et al., 2015), and other protozoan species such as *Trypanosoma* (Wagner and So, 1992). The diversity of ecDNA is exemplified in the reported sizes that range from a few hundred base pairs to megabase pair molecules (Dennin, 2018; Paulsen et al., 2018b). Although their size and composition vary depending on the organism, these elements are likely to facilitate rapid adaptation.

**FIGURE 1:**
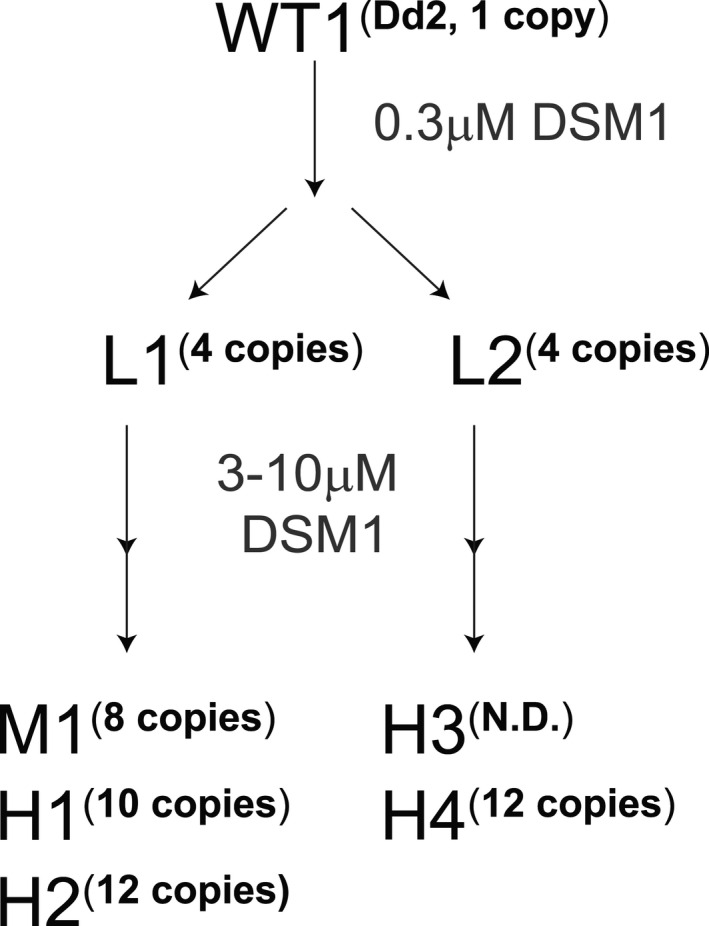
Summary of DSM1‐resistant parasite clones used in this study. Wild‐type*(*WT1, Dd2)*P. falciparum*was selected with DSM1 in two steps; the first step selected for low‐level (L) resistant parasites and the second step selected for moderate‐ (M) or high‐level (H) resistant parasites.*Dhodh*amplicon copy numbers (copies) were determined by qPCR; EC_50_values are as follows: L1 (1 µM), L2 (1 µM), M1 (7 µM), H1 (62 µM), H2 (85 µM), H3 (36 µM), and H4 (49 µM). All values were previously reported in and clone names adapted from (Guler et al.,^2013^). Not shown: Wild type 2 (WT2) represents Hb3 (0.1 µM, no resistance)

*Plasmodium falciparum*, the deadliest malaria parasite, killed ~405,000 people in 2018 (World Health Organization, 2019). This organism is known for its adaptability, both in response to the human immune system (Scherf et al., 2008) and drug treatment (Qidwai, 2020; White, 2004). However, the presence of ecDNA may contribute to rapid adaptation in *Plasmodium*, though such elements have not been reported to date. During our studies of parasites that were selected in a step‐wise manner with the novel antimalarial, DSM1 (Guler et al., 2013), a few key observations led us to hypothesize that ecDNA may be present in highly resistant *P. falciparum*. First, we observed genomic amplifications that included the target gene, *dihydroorotate dehydrogenase* (termed *dhodh* amplicon), and these resistance‐conferring amplicons rapidly increased with elevated antimalarial pressure. Second, the genomic position of the amplified unit was precisely conserved during subsequent selections, providing evidence that they were not generated de novo at every step. Lastly, the resistance level, although correlated, was not directly proportional to the detected amplicon copy number. Two DSM1‐resistant clones exhibited ~10 amplicon copies as detected by quantitative PCR (qPCR), but one of them (termed H1 for high level resistance) showed ~10‐fold higher EC_50_ than the other (termed M1 for moderate resistance, Figure 1). Overall, these observations indicated the possibility of an independent DNA element that contributes to resistance, which can be rapidly increased or decreased in order to balance the benefit with fitness effects.

Upon further study of this experimental system, we identified ecDNA in highly DSM1‐resistant *P. falciparum* parasites. We used an electrophoresis‐based purification scheme combined with highly sensitive DNA analysis methods to detect resistance‐conferring genes outside of the chromosomal genome. We also employed enzymatic digestions to determine the structure of the multiple forms of ecDNA. Finally, through deep sequencing of gel‐purified material, we discovered both conserved and unique ecDNA sequences. The carriage of resistance genes on ecDNA has wide‐reaching implications, since it provides a new target to limit the development of resistance. Understanding how these ancillary forms of DNA are generated and their function holds high importance for our understanding of adaptation by pathogenic organisms.

## 2 RESULTS

### 2.1 EcDNA is identified in high‐level DSM1‐resistant parasites

**FIGURE 2:**
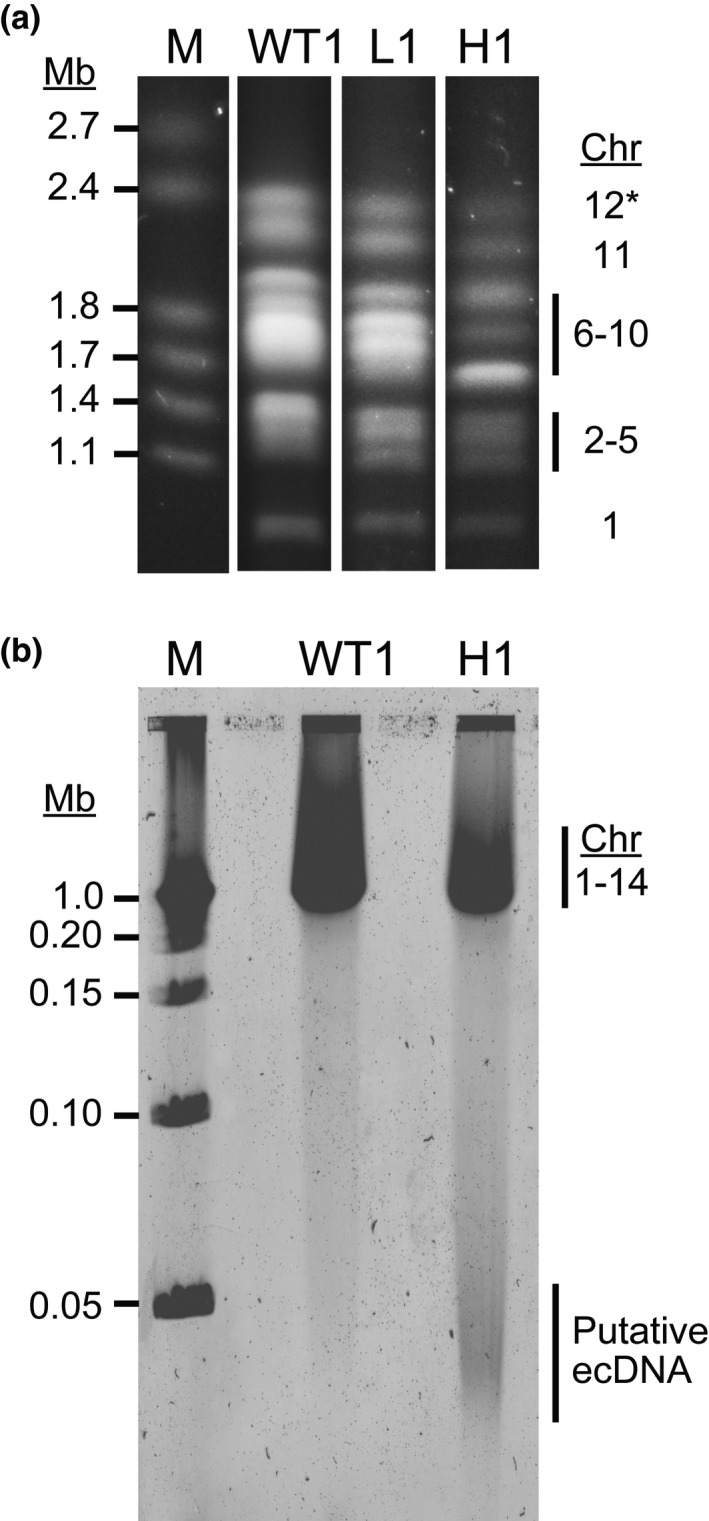
Pulse field gel electrophoresis reveals chromosomal shifts and aberrant DNA elements in highly resistant parasites. (a) Wild‐type (WT1:Dd2), low‐ (L1), and high‐level resistant parasites (H1) were embedded in agarose plugs, run on PFGE (50 hr, 3V/cm, 250–900 sec switch rate), stained with ethidium bromide, and imaged on a UV transilluminator. M, marker (1–3.1 Mb, BioRad 170‐3667). *Although not visualized in this blot, chromosomes 13 and 14 ran above chromosome 12 and appeared as expected in all clones. (b) Parasites were treated as in panel a prior to the PFGE run (17 hr, 6V/cm, 1–10 sec switch rate), stained with SYBR Safe DNA stain, and imaged using the Typhoon 9410 Variable Mode Imager. M, marker (0.05–1 Mb, BioRad 170‐3635); Chr, chromosome

In our initial study of drug resistance mechanisms in *Plasmodium*, we selected for resistant parasites using multiple levels of DSM1 selection (Guler et al., 2013) (see schematic in Figure 1 showing the relationship between L, M, and H clones). In order to investigate the physical nature of *dhodh* amplicons, we assessed the chromosomal pattern of DSM1‐resistant clones using pulse field gel electrophoresis (PFGE). Chromosomes 6–10 are similar in size (predicted to be 1.4–1.7 Mb based on the 3D7 genome (PlasmoDB, [Aurrecoechea et al., 2009]) and a shift of chromosomes in this region was evident in the resistant clones (Figure 2a). Prompted by this result, we ran the gels under a number of additional PFGE conditions. Those that allowed visualization of the broadest range of DNA sizes revealed an aberrant DNA element present in the H1 clone (Figure 2b). This “gel‐competent” ecDNA element ran independently from intact chromosomes as a heterogeneous smear regardless of PFGE run parameters (see various run conditions in *Experimental Procedures*).

### 2.2 Chromosomal amplicons do not account for high‐level resistance

**FIGURE 3:**
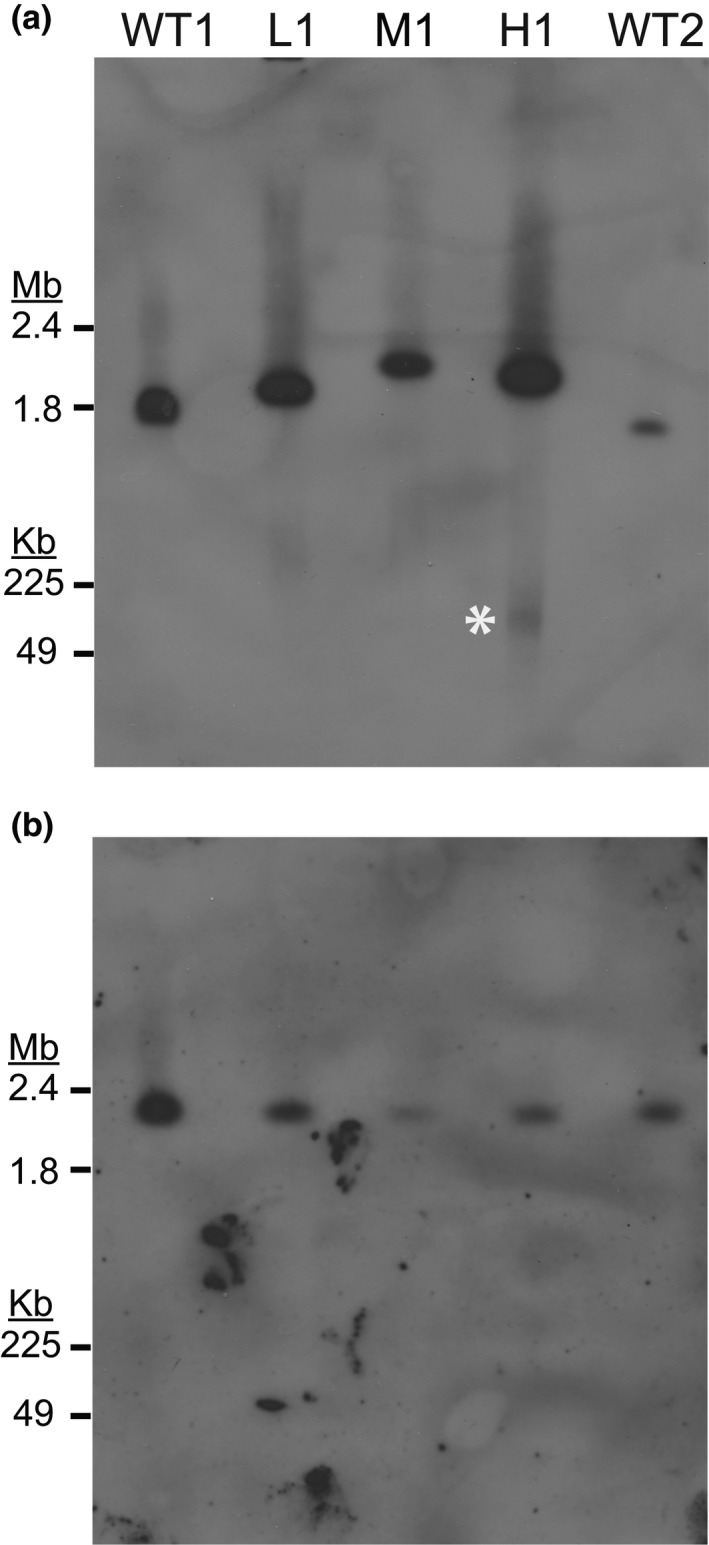
Southern blot analysis detects the resistance gene on both chromosomal and extrachromosomal DNA. Wild‐type (WT1:Dd2, WT2:HB3), low‐ (L1), moderate‐ (M1), and high‐level resistant parasites (H1) were embedded in agarose plugs, run on PFGE (50 hr, 3V/cm, 250–900 sec switch rate), transferred to a membrane, and probed for specific genomic loci. For all blots, the entirety of the gel is shown below the loading well and DNA size was determined with a 1–3.1 Mb marker (upper gel region, Bio‐Rad 170‐3667) and a 0.05–1 Mb marker (lower gel region, Bio‐Rad 170‐3635). (a) Southern blot probed for the*dihydroorotate dehydrogenase*gene (*dhodh*, Table 1) that is contained within the amplicon and confers DSM1 resistance (Guler et al.,^2013^). The expected size of WT1 chromosome 6 is 1.35 Mb and WT2 chromosome 6 is 1.33 Mb (PlasmoDB [Aurrecoechea et al.,^2009^]). Film exposure time: 8 hr. White asterisk, gel‐competent ecDNA. (b) Southern blot probed for single copy gene on chromosome 11 (Table 1). Analysis of an additional housekeeping gene was also performed on a different blot (single copy gene on chromosome 7, Table 1, FigureS2b). The expected size of WT1 and WT2 chromosome 11 is ~2 Mb (PlasmoDB [Aurrecoechea et al.,^2009^]). Film exposure time: 7 hr

Before we investigated the putative ecDNA, we further assessed the chromosomal amplicons using Southern blot analysis with probes for gene sequences within the *dhodh* amplicon, outside the amplicon on the same chromosome (chromosome 6 single copy gene, CEP76), as well as those on different chromosomes (chromosome 11 single copy gene, H protein; and chromosome 7 single copy gene, seryl‐tRNA synthetase, Table 1). Using the *dhodh* amplicon probe, we determined the relative sizes of chromosome 6 in wild‐type and resistant parasites (see summary in Table 2). Chromosome 6 from low‐ (L1) and moderate‐ (M1)level resistant clones, which are expected to have ~4 and ~8 copies of the amplicon relative to the rest of the genome, respectively (as determined by qPCR on genomic DNA, Figure 1 and Table 2), migrated with roughly the expected increase in size on PFGE (Figure 3a). However, chromosome 6 from the H1 clone, which is predicted to harbor ~10 relative copies of the amplicon, ran well below the predicted size of >2.5 Mb on PFGE (Table 2). In fact, the observed H1 chromosome 6 band ran below that from the M1 clone, with fewer predicted amplicon copies, and similar to the L1 clone (Table 2, Figure 3a). This latter result was verified with “in‐plug” restriction digestion followed by PFGE at ~50 kb resolution and Southern blot analysis; using BamHI alone (Figure S1a), which cuts only outside of the chromosomal *dhodh* amplicons in L1 and L1‐derived clones (i.e., H1 and H2, Figure 1), we detected a fragment size that indicates a similar number of amplicon copies in both resistance levels (~3 to 4 copies, Table S1, Figure S1b, lanes 3, 5, 11, and 17). Given our previous qPCR results (~10 to 12 *dhodh* amplicons, Figure 1, [Guler et al., 2013]), this finding indicates that a *minority* of amplicons sit in the home chromosomal location (~30% of the predicted total number of amplicons). In a second condition where a double digestion was performed with enzymes that flank and cut once in the *dhodh* amplicon (BamHI and NheI, respectively, Figure S1a), we detected fragments that were of the expected size to represent single amplicon units originating from the chromosome (Table S1 and Figure S1b, lanes 4, 6, and 12).

**Table 1:** Details of primers used in Southern blot analysis and Digital Droplet PCR (ddPCR)

Method	Probe Name	PlasmoDB Gene ID	Gene Name	Chr	CN	Sequence
Southern blot	*dhodh* amplicon	PF3D7_ 0603300	*dihydroorotate dehydrogenase*	6	Multi	F‐ TTGGTACCATAACCCCAAGG R‐ CCCTCCTGATGCAATAATGG
	Chr. 6 single copy gene	PF3D7_ 0,603,800	*centrosomal protein CEP76, putative*	6	Single	F‐ TGGGGGTCTTCTTCATCTTG R‐ ATACGTTCGTCGGATTTCCA
	Chr. 7 single copy gene	PF3D7_ 07177700	*seryl‐tRNA synthetase*	7	Single	F‐ TGCCGAACTTGATGACTTTG R‐ TGCGTTGTTTAAAGCTCCTG
	Chr. 11 single copy gene	PF3D7_ 1132900	*H protein*	11	Single	F‐ AAGTGCTTCTTCCCAGTTGTG R‐ CCCTTGCCCTTTATTTTCAA
ddPCR	*dhodh* amplicon	PF3D7_ 0603300	*dihydroorotate dehydrogenase*	6	Multi	F‐ TCCATTCGGTGTTGCTGCAGGATTT GAT^a^ R‐TCTGTAACTTTGTCACAACCCATATT A^a^ P:56FAM/CATTATTGCATCAGGAGGGA/MGBNFQ
	Chr. 7 Single copy gene	PF3D7_ 0717700	*seryl‐tRNA synthetas*e	7	Single	F‐ GGAACAATTCTGTATTGCTTTACC^a^ R‐ AAGCTGCGTTGTTTAAAGCTC^a^ P:VIC/ACATGAAGAAATGATACAAACA/3MGBEc
	Chr. 8 single copy gene	PF3D7_0831700	*heat shock protein 70‐X*	8	Single	F‐ GAATCGGTTTGTGCTCCAAT^b^ R‐ CAACTGTTGGTCCACTTCCA^b^ P:5HEX/AGCAGGAATGCCAGGAA/3MGBEc
	*mt‐cyb*	mal_mito_3	*cytochrome* b	Mit	Multi	F‐ AGCAAGTCGATATACACCAGATG R‐ CAAGAGAAGCACCTGTTGCG P:56FAM/AAGAGAATTATGGAGTGGATGGTGTTT/3MGBEc

Note

Chr, chromosome; CN, relative copy number; F‐, forward primer; P‐, probe; R‐, reverse primer.

^a^ Original primer reference (Guler et al., 2013).

^b^ Original primer reference (Ngwa et al., 2013).

**Table 2:** Comparison of expected and observed band sizes from Southern blot analysis using the *dhodh* probe

Resistance level	Clone	Average # of amplicons from qPCR^a^	Predicted increase in size^b^ (Mb)	Expected size (Mb)	Observed size (Mb)
Wild type	WT1	1	—	1.4^c^	~1.8
	WT2	1	—	1.3^c^	<1.8
Low	L1	4	0.292	~2.1	1.8–2.4
Moderate	M1	8	0.584	~2.4	1.8–2.4
High	H1	10	0.730	~2.5	1.8–2.4^d^
	H4	12	0.876	~2.6	1.8–2.4

^a^ These values were derived from qPCR on purified genomic DNA and previously presented in (Guler et al., 2013). They likely represent the total number of amplicons present per parasite (chromosomal + ecDNA).

^b^ Number of amplicons (determined by qPCR) x amplicon size (Guler et al., 2013).

^c^ The size of WT1 (Dd2) and WT2 (Hb3) chromosome 6 was retrieved from PlasmoDB (Aurrecoechea et al., 2009).

^d^ H1 chromosome 6 appears smaller than that from M1, despite an increased number of amplicons. Next Generation Sequencing revealed a ~10 kb deletion on chromosome 6 in a related clone (H2, Guler et al., 2013), but this does not account for the size difference.

### 2.3 Multiple ecDNA elements are present in resistant parasites

Using Southern blot analysis to further investigate the origin of ecDNA in high‐level resistant H clones, we determined that the gel‐competent ecDNA was only detected when using the *dhodh* amplicon probe (Figure 3a and Figure S2). We did not detect this ecDNA element when probing for two genes located on chromosomes 7 and 11 (Figure S2b and Figure 3b, respectively). As a result of these findings, we ruled out general genome degradation as the source of the observed “smear” and concluded that the gel‐competent ecDNA is derived from amplified regions of the genome.

During our Southern blot analyses, we routinely observed DNA remaining in the well of the PFGE gel (Figures S2 and S3A). Since previous studies found that circular DNA of ≥30 kb in size is readily trapped in the loading well and the compression zone immediately below (Cole and Tellez, 2002; Gurrieri et al., 1999; Khan and Kuzminov, 2013; Khan and Kuzminov, 2017; Turmel et al., 1990), we asked whether larger sized ecDNA elements are present as well. Indeed, if we used a specialized grade of agarose that allowed larger DNA fragments to enter the gel (megabase grade agarose), we detected *dhodh* amplicon‐specific supra‐chromosomal DNA in H1–4 clones (Figure S3b,c, white asterisks). This observation suggested that there is a second form of ecDNA in H clones that is “gel‐incompetent” under *standard* PFGE conditions (i.e., use of pulse field grade agarose).

From a long exposure Southern blot, we observed that some fraction of gel‐competent ecDNA remained detectable after restriction digestion of H clones (Figure S1c, lanes 5, 6, and 9‐12, white asterisks). This persistence following digestion is likely due to the random fragmented nature of the gel‐incompetent ecDNA, which reduces the chance that a restriction site is present on each individual fragment (see depiction in Figure S1a). Importantly, this persistent species was not detected when DNA from the H1 clone was probed for a single copy gene on chromosome 6 that sits outside of the amplicon (Figure S1c, lanes 13‐18), indicating that the migration patterns of ecDNA elements are not a misinterpretation of anomalous amplicon‐containing chromosome 6. Additionally, we did not detect evidence that gel‐incompetent ecDNA was digested (i.e., increased band intensities or additional material unique to H clones). While replicating mitochondrial genomes and transfected episomes entrapped in the PFGE loading well appear to be impacted by restriction digestion (O’Donnell et al., 2001; Weissig and Rowe, 1999), our observation may be due to either methodological differences between studies (i.e., DNA purification prior to agarose immobilization vs. immobilization of intact parasites) or biologically relevant differences between chromosomal DNA versus ecDNA (i.e., distinct methylation patterns alter cutting of ecDNA by methylation sensitive enzymes such as NheI).

### 2.4 EcDNA elements are enriched in the resistance‐conferring gene

**FIGURE 4:**
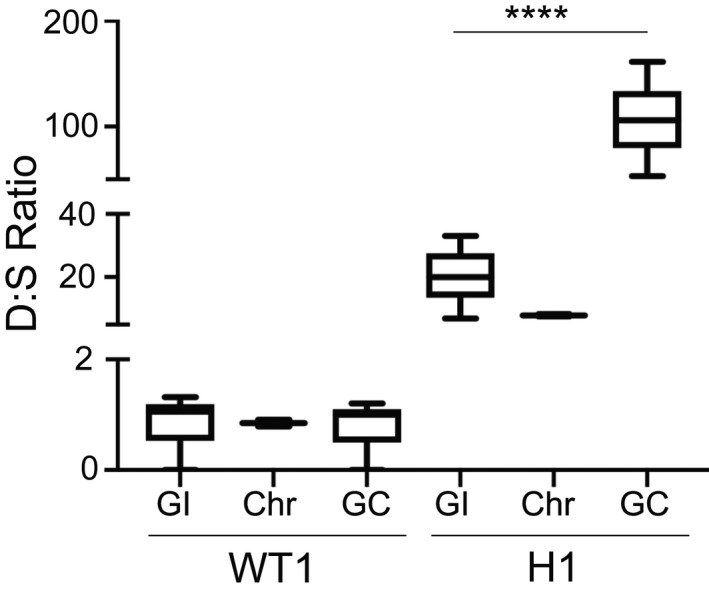
Droplet Digital PCR shows the enrichment of the resistance gene in extrachromosomal DNA. Following PFGE (as in Figure 2b), specific regions of the agarose gel were excised, DNA was purified, and assessed by Droplet Digital (dd)PCR for specific genomic loci. PFGE gel regions included the loading well with gel‐incompetent DNA (GI), the chromosomal (Chr) region with megabase‐sized chromosomes 1–14, and the lowest region that harbors gel‐competent DNA (GC) that runs between 50 and 200 kb. D:S Ratio, dihydroorotate dehydrogenase gene copies (*dhodh,*denoted as D, the resistance gene contained within the amplicon) divided by single copy gene copies (from chromosome 11, denoted as S, Table 1), as quantified by the Quantasoft software. The average D:S ratio measured in the WT1‐GI, Chr, and GC regions are 1.1, 0.9, and 1.0, respectively. The average D:S ratio (line in the center of the box) measured in the H1‐GI, Chr, and GC regions are 20, 7.9, and 106, respectively. Expected*dhodh*amplicon copy numbers for different clones are shown in Figure 1. ****p < .0001. Error bars represent Poisson confidence intervals (upper hinge, 75th percentile; lower hinge, 25th percentile);*N* = 3 per group

Traditional methods such as cesium‐chloride centrifugation and Hirt extraction were not effective in isolating ecDNA away from chromosomal DNA (Figure S4). Therefore, we took advantage of the electrophoretic separation of these elements from the chromosomes and purified them directly from the PFGE agarose gels. From this point on, we compared results from WT1 parasites with H clone parasites (i.e., H1) because ecDNA was most prominent at this resistance level. Following purification, we used Droplet Digital (dd)PCR to quantitatively measure enrichment of the resistance‐associated gene from three different gel regions of interest: the loading well (location of gel‐incompetent ecDNA), the chromosomal region (location of previously described tandem amplicons), and the smear (location of gel‐competent ecDNA). This method was particularly useful for our studies for several reasons: (a) the yield of ecDNA from the PFGE gel was below levels required to run conventional qPCR, (b) ddPCR assays can be multiplexed to accurately assess multiple loci within a single reaction, and (c3) droplet partitioning diluted contaminating material from purifications (*i.e.,* agarose). Using this approach, we calculated a “D:S ratio” between resistance‐conferring (*dhodh,* D) and single copy genes (*seryl‐tRNA synthetase*, S). For wild‐type samples, we observed a consistent D:S ratio of ~1, with no additional amplicons detected in either the extracted gel or loading well regions (Figure 4). For resistant parasites, we predicted that samples having higher levels of ecDNA should yield D:S ratios greater than the number of amplicon copies present in chromosomal DNA. Indeed, while gel‐incompetent and gel‐competent ecDNA elements yielded mean D:S ratios of 20 and 106, respectively (or 20‐ and 106‐fold enrichment of *dhodh* over *seryl‐tRNA synthetase*), chromosomal DNA yielded a mean D:S ratio of ~8 (Figure 4). This latter estimate is higher than those from Southern blot analysis (~3 to 4 *dhodh* copies, Table S1 and Figure S1b) perhaps owing to contamination by gel‐competent ecDNA. Thus, PFGE separation combined with highly sensitive ddPCR analysis shows that the ecDNA elements are enriched in the resistance‐conferring gene.

### 2.5 Gel‐incompetent ecDNA elements are complex in structure

**FIGURE 5:**
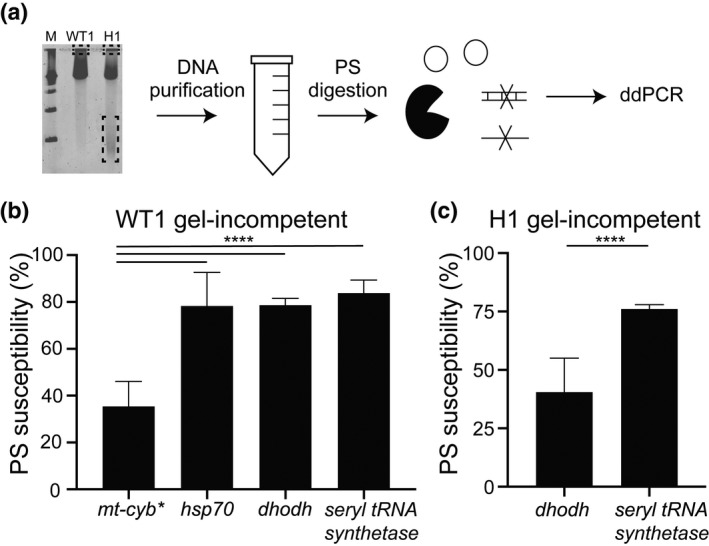
Exonuclease digestion indicates structural conservation between the complex mitochondrial genome and extrachromosomal DNA. (a) Following PFGE (as in Figure 2b), specific regions of the agarose gel were excised (as in Figure 4), DNA was purified, pre‐treated with a frequent cutting restriction enzyme (not depicted), digested with the PS exonuclease, and assessed by Droplet Digital (dd)PCR for specific genomic loci (either single or multi‐copy, Table 1). For simplicity, circular molecules represent both complex and circular DNA elements. (b and c) The PS susceptibility of each genomic locus was determined by quantifying DNA‐containing droplets using ddPCR; the % PS susceptibility (or level of DNA degradation) was calculated by dividing the number of loci‐positive droplets after digestion by the number of loci‐positive droplets before digestion and multiplying by 100. Single copy genes:*heat shock protein*(*hsp70*) and*seryl‐tRNA synthetase*; multi‐copy genes: mitochondrial*cytochrome b*(mt‐cyb*)and*dihydroorotate dehydrogenase*(*dhodh*, Table 1). *, although the majority of*P. falciparum*mitochondrial genomes are linear, complex structural forms are entrapped in the PFGE loading well. Error bars denote standard error; ****p < 0.0001. (b) DNA derived from the loading well of WT1 (Dd2, gel‐incompetent DNA, N = 3). (c) DNA derived from the loading well of the high‐level resistant clone H1 (gel‐incompetent DNA, N = 3)

To gain additional structural insight into the different ecDNA elements isolated from the gel, we utilized the Plasmid Safe (PS) ATP‐dependent DNase. This exonuclease preferentially digests linear DNA, while leaving circular DNA mostly intact. To examine its activity on *Plasmodium* DNA in general, we PS‐treated genomic DNA purified from cell lysates, linear chromosomal DNA isolated from gels, as well as complex structural forms of the mitochondrial genome that are also entrapped in the gel loading well. All samples were restriction digested with a frequent cutting enzyme in order to enhance the PS digestion of linear double‐stranded DNA (see *Experimental Procedures*). Following PS degradation, we evaluated the remaining material with ddPCR (Figure 5a); in addition to *dhodh* (on chromosome 6), we probed for a gene located on the complex/circular mitochondrial genome (*cytochrome b*), as well as genes that are located on linear chromosomes (*seryl‐tRNA synthetase* and *hsp70‐X* on chromosomes 7 and 11, respectively, Table 1). We found that the PS enzyme broadly degraded the genes from linear chromosomes from either the wild‐type or resistant clones from a variety of sources (i.e., genomic DNA: Figure S5a,c, the PFGE loading well: Figure 5b, or PFGE‐purified chromosomes: Figure S5b,d, mean of 87% across all samples). In contrast, the PS enzyme left the mitochondrial genome predominantly intact (mean of 36% degradation of the *cytochrome b* locus (*mt‐cyb*, Figure 5b). The PS enzyme also displayed limited digestion of gel‐incompetent ecDNA from the H1 loading well (average of 42% degradation at the *dhodh* locus, Figure 5c). This latter result is not due to a general lack of PS digestion since assessment of a chromosomal gene in the same sample showed high levels of degradation (average of 76% degradation at the *seryl‐tRNA synthetase* locus, Figure 5c). Although we were only able to assess the impact of the PS enzyme in a single experiment, we observed almost complete digestion of the gel‐competent ecDNA element (93% degradation at the *dhodh* locus, Figure S6). From these experiments, we concluded that H clone‐derived gel‐incompetent ecDNA is mostly complex/circular due to PS protection while gel‐competent ecDNA is likely linear due to high levels of PS susceptibility.

### 2.6 Gel‐incompetent ecDNA contains both conserved and unique sequence

Finally, we employed deep sequencing to search for unique aspects of H clone‐derived gel‐incompetent ecDNA. Due to low DNA yields from PFGE purification, it was necessary to amplify purified material prior to sequencing. In an attempt to enrich for ecDNA further, we PS‐treated a subset of samples before this amplification step. However, the overall AT‐content of reads from these samples as well as the failure to map to the *P. falciparum* genome (Table S2) indicated that contaminating bacterial genomes were preferentially amplified under these conditions. This has been reported with whole‐genome amplification methods due to a contribution of bacterial products by amplification reagents (Liu et al., 2020) and is likely exacerbated in our experiments due to low PS susceptibility of circular bacterial genomes. Given this result, we proceeded with analysis of samples that were not pre‐treated with PS before amplification (see basic read mapping statistics in Table S3).

Base mapping accuracy (i.e., MapQ scores) and the mean library insert size of the sequenced material was equivalent across the non‐amplified H1 genomic DNA sample and two amplified well‐derived samples (H1 and WT1). Consistent with the bacterial contamination of amplification reagents (see above), we identified more contaminating bacterial reads in the well material than the genomic DNA sample (Table S3). Conversely, the well‐derived samples exhibited lower levels of human read contamination (Table S3), which is likely due to electrophoretic depletion of degraded human DNA from in vitro culture. After the removal of non‐*Plasmodium* reads, the AT‐content of reads from all samples were around the expected value for the *P. falciparum* genome (~80% (Gardner et al., 2002), Table S3).

Reads from the amplified well‐entrapped samples aligned across the majority of the *P. falciparum* genome (both H1 and WT1, >70% of the genome was covered by >1 read, Table S3). This result is not surprising given that (a) chromosomal sequence is detected in the loading well during ddPCR assays (i.e., single copy genes on chromosomes 7 and 11) and (b) whole‐genome amplification using multiple displacement amplification (MDA) is effective at amplifying very low amounts of DNA (Hosono et al., 2003; Wang et al., 2009). However, despite similar total read counts in H1 genomic DNA and gel‐incompetent samples (Table S3), reads from the gel‐incompetent sample aligned to less of the genome with high coverage levels (Figure S7, ~50% less of the genome is covered by >8‐reads); this discrepancy is either due to a bias in MDA amplification or the limited representation of the genome in the well. Furthermore, we detected a ~100‐fold enrichment of the mitochondrial genome in the H1 gel‐incompetent material over the nuclear genome compared to ~30‐fold enrichment in the matched H1 genomic DNA sample (Table 3). Read‐based enrichment of the mitochondrial genome in the loading well agrees with our ddPCR analysis and previous reports (Preiser et al., 1996).

**Table 3:** Summary of coverage enrichment at known copy number variations

Samples	Chromosome 6 coverage*			Mitochondrial genome coverage		Chromosome 5 coverage*		
	Chr 6	*dhodh* amplicon	CN	*mt‐cyb*	CN^a^	Chr 5	*mdr1* amplicon	CN
H1 genomic DNA	8.8x	79.2x	**9**	288.0x	**30**	9.0x	29.3x	**3**
H1 gel‐incompetent DNA^b^	5.8x	356.4x	**61** ^c^	712.0x	**98**	5.3x	14.0x	**3**
WT1 gel‐incompetent DNA^b^	1.5x	1.8x	**1**	44.8x	**24** ^d^	1.6x	3.5x	**2**

Note

CN, relative copy number (calculated by dividing mean coverage of the amplicon by the coverage of the remainder of the chromosome); *dhodh*, *dihydroorotate dehydrogenase*; published CN = 8–10 (Guler et al., 2013)*; mt‐cyb*, mitochondrial *cytochrome b*; published CN = 20–150 (Lane et al., 2018); *mdr1*, *multidrug resistance protein 1*, published CN = 2–3 (Triglia et al., 1991).

*Reads were aligned to the WT1 (Dd2) reference genome using BWA‐MEM and chromosomes (chr) 5 and 6 coverage was calculated as the mean across the chromosome, excluding amplified regions.

^a^ Copy number of mitochondrial genome relative to nuclear genome.

^b^ These samples were isolated from the loading well of a PFGE gel and amplified using a DNA amplification kit to generate enough material for sequencing.

^c^ This amplicon includes the super‐peak region detailed in Table S4. Without this region, the estimated CN is ~15 copies.

^d^ The CN in the mitochondrial genome relative to the nuclear genome in WT1 genomic DNA is estimated to be ~15 based on qPCR for cytochrome b (Pholwat et al., 2017) and whole‐genome sequencing (Liu et al., 2020).

Bold indicates comparison across categories.

**FIGURE 6:**
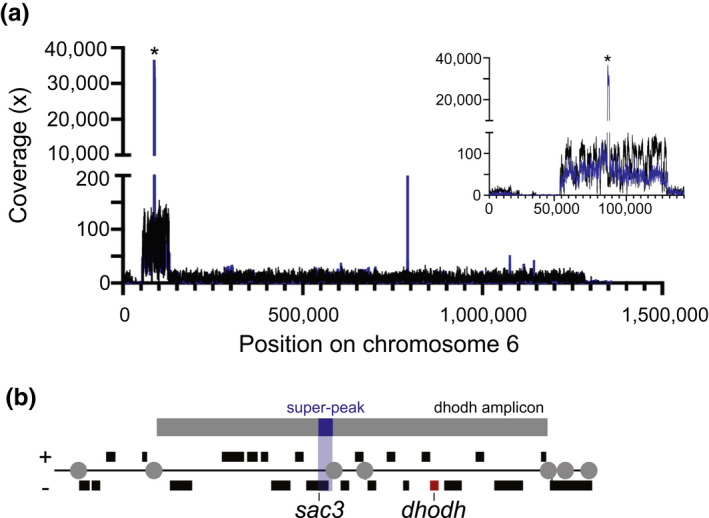
Deep sequencing shows conservation of amplicon boundaries and ecDNA‐specific sequence. Following PFGE (as in Figure 2b), the loading well was excised from WT1 (Dd2) and the highly resistant H1 clone (containing gel‐incompetent DNA), DNA was purified, amplified, and sequenced on the Illumina MiSeq platform. H1 gDNA and was also sequenced for comparison. (a) Read coverage across chromosome 6 from H1 gDNA (black line) and H1 gel‐incompetent DNA (blue line) was visualized using Integrative Genomics Viewer Software (IGV 2.4.10). Right inset, zoom of the*dhodh*amplicon. *Dramatic overrepresentation of a <1 kb region partially spanning the*sac3 gene*(PlasmoDB ID: PF3D7_0602600) in the H1 gel‐incompetent sample (termed super‐peak due >30,000‐fold coverage, Table S4). (b) The relative gene locations (black boxes) and the super‐peak location (blue shaded region) within the full*dhodh*amplicon (gray box, top) adapted from (Guler et al.,^2013^). Red box,*dhodh*gene; gray circles, location of long A/T tracks involved in CNV formation; black boxes, genes with ±orientation

When comparing read coverage across the *dhodh* amplicon relative to that from the rest of chromosome 6, we observed a high level enrichment in the gel‐incompetent material (mean of ~60‐fold, Table 3) and conservation of the amplicon boundaries with those from genomic DNA (Figure 6a). Additionally, we discovered an AT‐rich (88.2%), 714 bp sequence within the *dhodh* amplicon that was dramatically over‐enriched in the H1 gel‐incompetent material. This “super‐peak” exhibited coverage levels of >30,000x, which is >5,000‐fold greater than the rest of chromosome 6 (Table S4). By excluding the super‐peak region from this analysis, we detect a similar number of *dhodh* amplicons as estimated by ddPCR (~15‐fold enrichment, Table 3). Initially, we suspected that the extremely high coverage at the super‐peak was due to an artifact of MDA amplification, which exhibits random bias across genomes of multiple organisms including *Plasmodium* (Chen et al., 2014; Liu et al., 2020). Indeed, other small regions of the genome were over‐amplified in the H1 well‐derived sample, although not to the same extent (mean of ~240‐fold, Table S5). However, the super‐peak region was not over‐represented in the amplified WT1 sample (Table S4). Analysis of discordant reads at this location revealed that the amplified sequence contributing to the super‐peak is arranged in a tandem head‐to‐tail orientation (Figure S8a,b), which is similar to the orientation of chromosomal copies of the *dhodh* amplicon (Guler et al., 2013) (Figure S8c). This pattern, however, is not evident at other over‐amplified regions across the genome (*data not shown*), likely because MDA creates randomly connected sequences, or chimeric reads, that occur due to template switching during high polymerase processivity (Lasken and Stockwell, 2007). Due to the extreme level of over‐amplification and evidence of its tandem orientation, the super‐peak may represent a sequence that was present at high levels prior to amplification steps and may, therefore, have biological significance. A targeted analysis of this region in non‐amplified sample is precluded by the extreme AT‐content of this region (~90%), which makes the design of specific PCR primers impractical and complicates our ability to demonstrate the physiological relevance of this sequence.

Finally, in order to understand whether the ecDNA phenomenon is restricted to the *dhodh* amplicon in H clones, we evaluated the level of other known copy number variations in the sequenced well‐derived material. From this analysis, we observed similar enrichment levels between genomic DNA and gel‐incompetent samples for *mdr1* amplicons located on chromosome 5 (expected: 2‐ to 3‐fold, (Triglia et al., 1991), observed: 2‐ to 3‐fold, Table 3). This finding suggests that ecDNA may be confined to situations where high numbers of amplicons are required for resistance and/or they are only detectable when under active selection.

## 3 DISCUSSION

We have identified ecDNA in DSM1‐resistant *P. falciparum* parasites. This is the first observation of endogenously derived ecDNA in any *Plasmodium* species following drug selection. Our use of both traditional and modern approaches facilitated our identification and direct study of ecDNA. Using electrophoresis‐based methods, we made a number of important observations about these molecules including their confinement to H clones and the existence of two distinct ecDNA elements (Figures 3 and 4). Importantly, we also determined that the majority of amplicons within a parasite are maintained on ecDNA; only ~30% of the predicted total amplicons sit in the home chromosomal location (Table S1 and Figure S1b). The existence of unstable ecDNA (Beverley et al., 1984; O’Donnell et al., 2002), alongside stable chromosomal amplicons, is consistent with our previous observation that most amplicons are lost without drug pressure and only a few persist long term (~2 to 3 copies, >6 months, [Guler et al., 2013]). We have high confidence in our gel‐based results due the observations that ecDNA elements were only observed in resistant parasites and not wild‐type parasites that are prepared in the same way and only detected with probes for the *dhodh* amplicon and not probes from other regions of the genome (Figures 2 and 3, Figures S1–S3). Our use of sensitive DNA analysis methods also confirmed this amplicon‐specificity of ecDNA (ddPCR, Figure 4) and revealed unique aspects of ecDNA sequence (deep sequencing, Figure 6). Although we cannot completely rule out alternative explanations, we have gathered strong evidence of ecDNA in drug‐selected *P. falciparum* parasites. Below, we summarize information on various characteristics of this novel ecDNA. Additionally, we discuss potential mechanisms for its generation and role in the biology of malaria parasites.

### 3.1 The two ecDNA elements and their relationship

**FIGURE 7:**
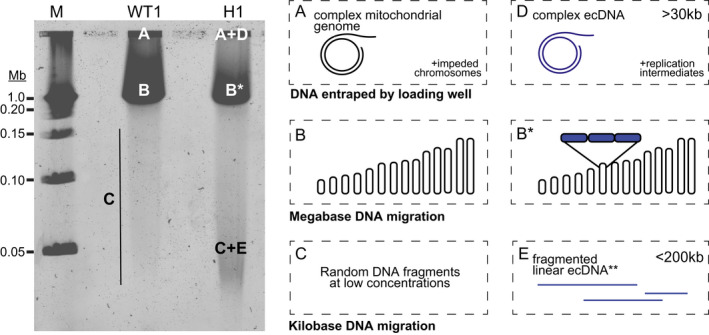
Summary of DNA structures predicted by agarose gel location and enzymatic analyses. (A) After PFGE, the loading well contains complex/circular forms of the mitochondrial genome and a small proportion of nuclear chromosomes and their replication intermediates. (B) The prominent band contains linear megabase‐sized chromosomes 1–14. Under these PFGE conditions, individual chromosomes are not resolved. (B*) In highly resistant parasites, the size of chromosome 6 varies due to the presence of tandem*dhodh*amplicons (blue boxes). (C) Kilobase‐sized nuclear DNA fragments migrate below the chromosomes; their size varies due to stochastic breakage during electrophoresis. (D) In highly resistant parasites, the loading well contains complex/circular forms of ecDNA and accompanying replication intermediates, which may contain stretches of fragile linear DNA. (E) The low migrating “smear” contains linear ecDNA elements that break from complex/circular forms during electrophoresis. WT1, Dd2; H1, high‐level resistant clone; M, marker

We first observed the gel‐competent ecDNA in H clones as a heterogenous smear of <200 kb that was enriched in the resistance‐conferring *dhodh* gene (Figures 2b, 3a, 4 and Figure S2). This observation, combined with our limited assessment of PS susceptibility (Figure S6), supports the linear, yet fragmented nature of this ecDNA element (Figure 7e). We identified another form of ecDNA in H clones that is distinct from the gel‐competent ecDNA element. Instead of entering the gel, this ecDNA form remains in the PFGE loading well (Figure 7d). This phenomenon, described as “entrapment,” was previously described during PFGE analysis of large (≥30 kb), circular or highly branched recombination intermediates from other organisms (Beverley, 1988; Gurrieri et al., 1999; Khan and Kuzminov, 2013; Khan and Kuzminov, 2017; Turmel et al., 1990) (Figure 7a). In *P. falciparum*, some forms of the mitochondrial genome are complex and also entrapped in the PFGE well (Preiser et al., 1996; Weissig and Rowe, 1999). Due to this characteristic, as well as its enrichment over the nuclear genome (Lane et al., 2018; Preiser et al., 1996; Vaidya and Arasu, 1987), we were able to use mitochondrial DNA as an endogenous control during our ecDNA studies. We showed that sequences from both complex molecules were enriched in the PFGE well material (Table 3). We also observed partial protection from PS degradation for both the mitochondrial genome and gel‐incompetent ecDNA, indicating potential structural conservation between the two molecules (Figure 5b,c).

Our studies show that both novel ecDNA elements are derived from the resistance amplicon and, therefore, they either share a common origin or one is generated from the other. Since linear DNA elements are not maintained in replicating parasites (O’Donnell et al., 2001; Williamson et al., 2002a; Wu et al., 1995), we propose that the observed gel‐competent ecDNA element is generated by breaking from the more complex gel‐incompetent element (Figure 7d,e). In preparation for PFGE analysis, parasites are embedded within agarose without any DNA purification steps. Therefore, DNA breakage occurs during electrophoresis. While we observed a low rate of breakage from intact chromosomes, this occurrence appears exacerbated for the large complex ecDNA molecule. Given the parallels with the structure of the mitochondrial genome mentioned above, it is possible that the gel‐incompetent ecDNA contains short stretches of fragile linear DNA (as described for the mitochondrial genome, [Preiser et al., 1996]). Random breakage across these regions can generate a heterogeneous mixture of sizes and explains the diffuse nature of the gel‐competent ecDNA.

### 3.2 ecDNA sequence; a mixture of conserved and unique

A number of techniques show that the gel‐incompetent ecDNA element shares major homology with the amplicon on chromosome 6 (Figures 5 and 6, Figure S3). Additionally, the chromosomal amplicon and the enriched ecDNA amplicon exhibit the same boundaries and orientation, indicating that the full ~70 kb *dhodh* amplicon is conserved (Figure 6a). Deep sequencing of the well‐derived material revealed a feature that was unique to ecDNA: a small region of the amplicon was highly overrepresented in gel‐incompetent H1 material (Figure 6a, termed the super‐peak). This region encompassed a portion of the upstream UTR and 5′ end of the gene for the SAC3 domain‐containing protein (PlasmoDB gene ID: PF3D7_0602600). Certain characteristics, including its excessive overrepresentation and the predicted head‐to‐tail orientation of the copies (Table S4 and Figure S8), suggest that the super‐peak has biological relevance. The AT‐richness, small size, and tandem arrangement of the super‐peak sequence is reminiscent of *Plasmodium* replication origins (~500 bp, >75% AT‐content) and centromeres (~2 kb, 97% AT‐content) (Agarwal et al., 2017; Gardner et al., 2002; Iwanaga et al., 2010; Matthews et al., 2018; Singh et al., 2003; Verma and Surolia, 2018). While previous studies correlated the stability of transfected episomes in malaria parasites with AT‐rich centromere‐like elements that increased efficiency of mitotic segregation (Iwanaga et al., 2012; Iwanaga et al., 2010; Verma and Surolia, 2018), more investigation is required to determine if these conserved characteristics impact ecDNA segregation.

### 3.3 Model of ecDNA generation and maintenance

**FIGURE 8:**
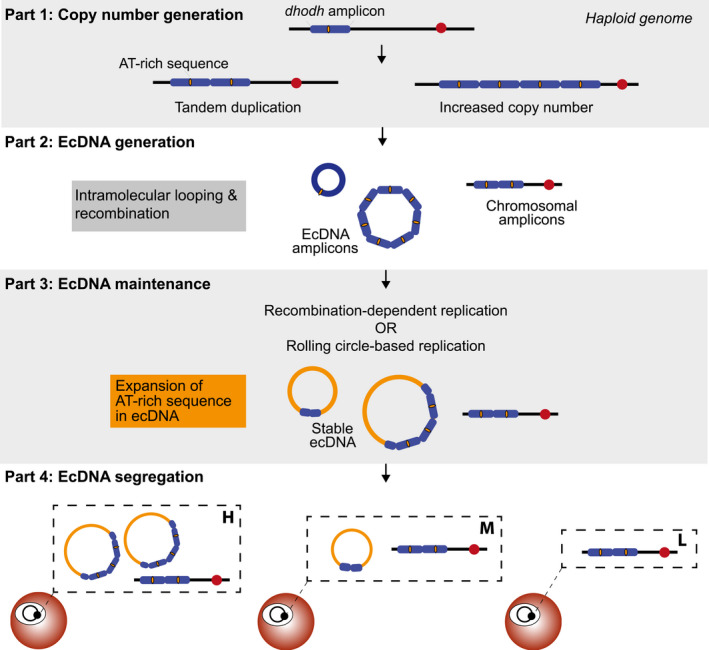
A model of ecDNA generation and maintenance in*Plasmodium falciparum*. Part 1 summarizes the paths to generate few to many tandem*dhodh*amplicons on chromosome 6 (blue boxes) under drug selection.*Dhodh*amplicons do not contain known centromere sequences (red circle) but they do potentially harbor a short, AT‐rich sequence in the center of the amplicon that is expanded in ecDNA molecules (yellow region). Note: the physiological relevance of this sequence has not yet been demonstrated. Part 2 depicts the generation of circular ecDNA by loop formation and recombination. Individual ecDNA molecules likely contain a range of amplicon numbers (although higher numbers may facilitate segregation) and some amplicon copies remain stable on chromosome 6. Part 3 highlights proposed mechanisms of replication that may contribute to maintenance of ecDNA. We speculate that the expansion of the short, AT‐rich sequence (yellow region, >5,000‐fold enrichment over other sequences, termed “super‐peak”) may occur during replication and contribute to ecDNA stability. Part 4. The presence of ecDNA dictates the level of resistance achieved by the parasites (H, high level resistance; M, moderate resistance; L, low level resistance). Unequal segregation of ecDNA into daughter parasites, even in a clonal parasite line, likely contributes to the growth phenotype observed in highly resistant parasites (Guler et al.,^2013^)

Many mechanisms, either replication‐dependent or independent, have been suggested to contribute to the generation of ecDNA (Paulsen et al., 2018a). We propose a model of ecDNA generation that integrates our previous studies on chromosomal amplicons (Guler et al., 2013; Huckaby et al., 2019) with the current experimental results (Figure 8). After the formation of amplicons (Figure 8, *Part 1*, left side), large stretches of homology between amplicons mediate recombination. This process can lead to the expansion of amplicons (as described in (Guler et al., 2013), Figure 8, *Part 1*, right side), as well as the formation of ecDNA (as described in Figure 8 *, Part 2*). For the latter, a commonly proposed mechanism involves loop formation followed by a homologous recombination event between repetitive sequences. This generates a circular ecDNA element as well as a healed chromosome and is consistent with a predominance of homologous recombination activity in the parasite (Kirkman et al., 2014).

In considering the maintenance of ecDNA, we once again draw on parallels with the mitochondrial genome. A recombination‐dependent mechanism is responsible for the replication of the mitochondrial genome and maintenance of transfected episomes in *P. falciparum* (O’Donnell et al., 2001; Preiser et al., 1996). A conserved mechanism may participate in the replication of ecDNA; moreover, D‐loop and/or rolling circle replication, as used for the *P. falciparum* apicoplast genome (Milton and Nelson, 2016; Williamson et al., 2002b), may also be acting. Either process would explain two prominent characteristics of *P. falciparum* ecDNA: (a) PFGE well entrapment due to the formation of massive complexes of single‐ and double‐stranded DNA (Viret et al., 1991) and (b) the heterogeneity of the smear due to the creation of varying lengths of linear tails during active replication (O’Donnell et al., 2001). Based on this supporting evidence, we propose that conserved pathways are also involved in ecDNA replication (see Figure 8, *Part 3*).

Once replicated, ecDNA must be segregated during cell division so that daughter cells can maintain their selective advantage (see Figure 8, *Part 4*). Although ecDNA shares close identity to a region of chromosome 6 (position 61,619‐158,072 [Guler et al., 2013]), this region does not contain a centromere (predicted position at 477,751‐482,751 [Hoeijmakers et al., 2012]). Other possibilities for segregation include direct tethering as has been reported in other organisms (Kanda et al., 2007; Kanda and Wahl, 2000; Sau et al., 2019), hitchhiking due to its large size as has been observed for *P. falciparum* episomes (O’Donnell et al., 2001), or use of repetitive regions as pseudo‐centromeres as we suggest for the super‐peak (see *ecDNA Sequence*, Figure 8, *Part 3*). In all of these cases, unequal segregation is likely. This characteristic has been observed for transfected episomes (Anderson et al., 2011) and could contribute to the growth phenotype observed for H clones (Guler et al., 2013); only the proportion of parasites that receive enough ecDNA survive in the highest levels of the antimalarial treatment (see Figure 8, *Part 4*).

### 3.4 Implications of ecDNA in malaria

In light of the identification of ecDNA in highly DSM1‐resistant parasites, it is important to consider whether ecDNA could contribute to resistance in other antimalarial contexts. Due to its effect on DNA synthesis, DSM1 inhibition likely causes replicative stress, which could trigger pathways that drive copy number variations and/or ecDNA generation (Arlt et al., 2009; Arlt et al., 2009; Bindra et al., 2004). However, copy number variations are not specific to this drug resistance model; amplicons are observed in parasites resistant to a range of antimalarials with a number of cellular targets (Anderson et al., 2009; Cheeseman et al., 2009; Cowman et al., 1994; Foote et al., 1990; Kidgell et al., 2006; Nair et al., 2008; Triglia et al., 1991; Wilson et al., 1989). Although we did not detect well‐entrapped ecDNA derived from the common *mdr1* amplicon in our experiments (Table 3), this could be influenced greatly by ecDNA abundance, presence and strength of selection, as well as the methods employed (single cell vs. population‐based). Improved detection methods, including new efforts to computationally identify circular DNA from sequencing data (Deshpande et al., 2019; Prada‐Luengo et al., 2019; Wu et al., 2019), hold promise for future studies of malaria ecDNA.

## 4 EXPERIMENTAL PROCEDURES

### 4.1 DSM1 and parasite clones

DSM1 is a triazolopyrimidine that specifically and potently inhibits the *P. falciparum* dihydroorotate dehydrogenase enzyme of pyrimidine biosynthesis (Phillips et al., 2008). DSM1‐resistant parasites were previously selected according to the scheme depicted in (Figure 1) (Guler et al., 2013). For this manuscript, we simplified the naming scheme to represent low (L), moderate (M), and high (H) levels of resistance (Figure 1).

### 4.2 Parasite culture

*P. falciparum* parasites were grown in vitro at 37°C in solutions of 2%–3% hematocrit (serotype A positive human erythrocytes) in RPMI 1640 (Invitrogen, Waltham, MA, USA) medium containing 28 mM NaHCO_3_ and 25 mM HEPES, and supplemented with 20% human type A positive heat‐inactivated plasma in sterile, sealed flasks, flushed with 5% O_2_, 5% CO_2_, and 90% N_2_ (Haynes et al., 1976; Rathod et al., 1992; Trager and Jensen, 1976). Cultures were maintained with media changes three times each week and sub‐cultured as necessary to maintain parasitemia below 5%.

### 4.3 Pulse field gel electrophoresis (PFGE)

Parasites embedded in agarose plugs for PFGE were made from synchronized trophozoite‐schizont *P. falciparum* parasites according to previous protocols (Hernandez‐Rivas and Scherf, 1997). Briefly, erythrocytes were lysed with 0.15% saponin (Acros Organic, Thermo Fisher Scientific, Waltham, MA, USA), parasites were washed in sterile phosphate buffered saline, and 5 × 10^8^ parasites/ml were resuspended in 1.6% certified low melting point agarose (Bio‐Rad Laboratories, Hercules, MA, USA) at 37°C (2.5 × 10^8^ parasites/ml final). Aliquots of 100 µl in total were cooled in plug molds before transferring to cell lysis buffer (10 mM Tris–HCl pH 8, 0.5 M EDTA, 1% sodium lauryl sarcosinate) for storage. To eliminate parasite proteins prior to PFGE analysis, parasite plugs were incubated with 2 mg/ml proteinase K (Thermo Fisher Scientific, Waltham, MA, USA) in cell lysis buffer at 37°C for two 24 hr incubations (with a buffer replacement between each). Plugs were loaded into the well of 1% pulse field certified agarose gel in 0.5X Tris/Boric acid/EDTA buffer (Bio‐Rad Laboratories) and run at 14°C on the PFGE‐DR system with PFGE DNA standards (Bio‐Rad Laboratories) at various conditions (see figure legends for marker information and running conditions for each gel). Completed gels were stained with either ethidium bromide (1 µg/ml, Bio‐Rad Laboratories), SYBR Safe DNA Gel Stain (1:10,000, Life Technologies, Carlsbad, CA, USA) or SYBR Gold Nucleic Acid Gel Stain (1:10,000, Invitrogen). Gels were visualized using either UV transillumination, Typhoon 9410 Variable Mode Imager (SYBR settings: fluorescence, Laser: blue (488nm), Emission filter: 520BP40), or Gel Doc XR + Gel Documentation (Bio‐Rad Laboratories).

### 4.4 DNA transfer, probe labeling, and Southern blot analysis

Following PFGE, ethidium bromide‐stained DNA embedded within the gels was nicked by UV treatment (60 mJ/600 × 100 µJ on a short wave/ 254 nm UV transilluminator). Gels were soaked in 0.4 N NaOH/1.5 M NaCl for 15 min prior to 24–48 hr alkaline transfer to nylon (Zeta‐Probe membrane, BioRad Laboratories). Membranes were rinsed in 2x saline sodium citrate and dried before probing. Candidate probe sequences were amplified from the WT1 (Dd2) *P. falciparum* genome using primers are listed in (Table 1). The PCR protocol was 94°C for 3 min, followed by 30 rounds of 94°C for 30 sec, 55°C for 1.5 min, and 68°C for 1.5 min and an extension step of 68°C for 5 min. PCR products were cloned into TOPO‐TA (Life Technologies) and confirmed by Illumina sequencing. Confirmed probes were labeled with digoxygenin (DIG): dNTP (1:6‐1:9) using the PCR DIG Probe Synthesis Kit (Roche Applied Science, Indianapolis, IN, USA) at the cycling conditions listed above.

For Southern blotting, membranes were pre‐hybridized in DIG Easy Hyb™ (Roche Applied Science) for 30 min at 42°C. Probes were denatured for 5 min at 95°C and immediately chilled in ice water prior to being added to pre‐warmed hybridization buffer at a concentration of 500–800 ng/ml. Following an 18–24 hr incubation at 42°C, probed membranes were washed twice for 5 min in 2X SSC/ 0.1% SDS at room temperature and then, twice more for 15 min in 0.5X SSC/ 0.1% SDS at 50°C. DIG‐labeled probe was detected on the membrane using the DIG Luminescent Detection Kit (Roche Applied Science) according to the manufacturer's instructions. Membranes were exposed to film and developed to visualize probe pattern. Prior to re‐probing, membranes were stripped twice for 15 min in 0.2 M NaOH/0.1% SDS at 37°C, and washed for 5 min in 2xSSC.

### 4.5 Restriction digestion prior to Southern blot analysis

Restriction sites were predicted from the 3D7 reference genome, but confirmed in sequencing reads from WT1 (Dd2), L1, and L2 clones generated during a previous study (Figure 1) (Guler et al., 2013). Restriction digests of the parasite plugs were performed as previously described (Hernandez‐Rivas and Scherf, 1997). Briefly, plugs that had been treated with cell lysis buffer and proteinase K (see *PFGE* section above) were washed three times in TE buffer (10 mM Tris–HCl pH8, 1 mM EDTA), treated with 1 mM phenylmethanesulfonylfluoride (Sigma‐Aldrich, St. Louis, MO, USA) for 2 hr at room temperature to inactivate proteinase K (Thermo Fisher Scientific) from the lysis step above, and washed three additional times in TE for 30 min. Plugs were then equilibrated in restriction endonuclease buffer (with bovine serum albumin) prior to incubation with 200 U/ml of enzyme (BamHI and/or NheI, New England Biolabs) at 37°C for 18 hr. Digestion was stopped by adding 50 mM EDTA prior to running on PFGE, transferring, and probing as above. The observation of a single band in standard film exposure times (30 min to 3 hr), and lack of a ladder of defined unit sizes, indicates that the digestion worked efficiently under these conditions.

### 4.6 Standard DNA isolation

Genomic DNA (gDNA) and other sources of DNA (see below) were purified prior to ddPCR as previously reported (Guler et al., 2013). Briefly, parasites were lysed with 0.1% L‐loril sarkosil (Teknova Inc, Hollister, CA, USA) in the presence of 200 μg/ml proteinase K (Thermo Fisher Scientific) overnight at 37°C. Nucleic acids were then extracted with phenol/chloroform/isoamyl alcohol (25:24:1), pH 7.8‐8.1 (Invitrogen) using Phase Lock Gel, Light tubes (5 Prime Theaetetus Inc, San Francisco, CA, USA). Following RNA digestion with 100 μg/ml RNAse A (Thermo Fisher Scientific) for 1 hr at 37°C, gDNA was extracted two additional times as described above, once with chloroform, and, then ethanol precipitated using standard method. DNA quantitation was performed using the Qubit double‐stranded DNA High Sensitivity kit per the manufacturer's instructions (Thermo Fisher Scientific).

### 4.7 DNA isolation from agarose gels

Regions of the gel with target DNA were excised from the PFGE agarose gels using a sterile razor and purified using a modified “freeze and squeeze” method (Thuring et al., 1975). The agarose block was placed in a sterile microcentrifuge tube with 500 µl of nuclease‐free water. Samples were heated for 65°C for 20 min and transferred to a −80°C freezer. Following an overnight incubation, the samples were thawed at room temperature and centrifuged for 30 min at 20°C at 17,000× *g* (instead of physically squeezing the gel between thumb and index finger, as performed in the original protocol). The supernatant was transferred to a new tube and DNA was purified using phenol/chloroform/isoamyl alcohol (Invitrogen) followed by ethanol using standard methods (see *Standard DNA Isolation* above). Purified DNA was quantified before use in downstream analyses by Qubit DNA quantitation (Qubit Fluorometer, Thermo Fisher Scientific, see *Genomic DNA Purification*).

### 4.8 Droplet Digital PCR and analysis

The PCR step of Droplet Digital (dd) PCR was performed using QX200™ EvaGreen^®^ ddPCR™ Supermix or ddPCR probe Supermix (Bio‐Rad Laboratories) with 5 pg–1 ng of DNA. The EvaGreen^®^ assays used 75–150 nM multiplexed primers (a single copy reference gene was run in the same reaction as the multi‐copy gene by varying the primer concentrations [McDermott et al., 2013; Miotke et al., 2014]) (Table 1). The PCR protocol for EvaGreen^®^ assays was 95°C for 10 min, followed by 39 rounds of 94°C for 30 sec and 60°C for 1 min, 4°C for 5 min and 90°C for 5 min. The TaqMan probe assay used 600 nM for VIC probes and 900 nM (final) for FAM probes multiplexed primers combined with a 50 µM (final) probe (McDermott et al., 2013; Miotke et al., 2014) (Table 1). The reactions also contained one of the following detection probes: *Cytochrome b* probe 5′‐56‐FAM/AAGAGAATTATGGAGTGGATGGTGTTT/3MGBEc‐3′, *Dihydroorotate dehydrogenase* (*dhodh*) probe 5′‐56‐FAM/CATTATTGCATCAGGAGGGA/3MGBEc‐3′, *heat shock protein 70 (hsp70*) probe 5′‐HEX/AGCAGGAATGCCAGGAA/3MGBEc‐3′, and *seryl‐tRNA synthetase* probe 5′‐5HEX/ACATGAAGAAATGATACAAACA/3MGBEc‐3′. The PCR protocol for probe‐based assay was 95°C for 10 min, followed by 40 rounds if 95°C for 30 sec and 60°C for 1 min. *Seryl‐tRNA synthetase* and *heat shock protein 70* and served as a single copy reference genes on chromosomes 7 and 8, respectively; *Cytochrome b* and *dihydroorotate dehydrogenase* served as multi‐copy genes (Table 1). Droplet generation (prior to PCR cycling) and fluorescence readings (post‐PCR cycling) were performed per the manufacturer's instructions. The majority of samples measured fluorescence from a minimum of 8,000 droplets, although droplet counts below this threshold were allowed for crude lysate material or digestion analysis. The ratio of positive droplets containing an amplified gene (*dhodh, D*) versus a single‐copy gene (*seryl‐tRNA synthetase, S*) (D:S) was calculated using the Quantasoft analysis software (BioRad Laboratories) and averaged between independent replicates. Poisson confidence intervals were provided by the software (QuantaSoft Version 1.7).

### 4.9 Digestion of linear DNA with PS ATP‐dependent DNase

To increase the efficiency of linear DNA degradation (as recommended by the manufacturer of Plasmid‐Safe), all samples were first treated with the frequent cutting Ndel restriction enzyme (10,000 units/ml each, New England Biolabs) at 37°C for 30 min. To remove residual protein and restriction buffer, samples were purified post‐digestion using phenol/chloroform/isoamyl alcohol (25:24:1), pH 7.8–8.1 (Invitrogen) followed by ethanol using standard methods (see *Standard DNA Isolation* above). Purified samples were then treated with 1 unit of PS ATP‐dependent DNase (Epicentre Technologies, Madison, WI, USA) per 10 µl reaction for 30 min at 37°C, followed by an inactivation step at 70°C for 30 min according to the manufacturer's instruction. Samples were evaluated by ddPCR for PS activity.

### 4.10 DNA amplification, Illumina sequencing, and analysis

To reach an optimal concentration for sequencing (>10 ng), we purified DNA from the PFGE loading well and amplified the DNA first using the Repli‐g Mini Kit (Qiagen, Germantown, MD, USA). Multiple displacement amplification (MDA)‐based methods such as Repli‐g have been employed previously for ecDNA studies (Jørgensen et al., 2015) and validated in *P. falciparum* as robust and accurate for genotyping (Nkhoma et al., 2020; Srisutham et al., 2020; Trevino et al., 2017; Wang et al., 2009). In addition to the Repli‐g amplified H1 well‐derived material, we selected the following controls for this experiment: (a) non‐amplified H1 gDNA for comparison of amplicon boundaries and enrichment levels (for both *dhodh* and *mdr1* amplicons) and (b) Repli‐g amplified WT1 well‐derived material for the enrichment of the mitochondrial genome and *mdr1* amplicons (compared to WT1 gDNA previously sequenced by our group, see Table 3).

The following components were added per 50 μl MDA reaction: 5 μl of template DNA (<10 ng), 5 μl of denaturation buffer, 10 μl of neuralization buffer, and a 30 μl reaction mix (of 1 μl of Repli‐g Phi 29 polymerase and 29 μl of Repli‐g Mini reaction buffer) as per the manufacturer's instructions. Samples were incubated at 30°C for 16–18 hr and heat inactivated at 65°C for 3 min. Genomic DNA samples from wild‐type and highly resistant parasites were purified as described above (see *Standard DNA Isolation*). The H1 gDNA used in these studies was not MDA‐amplified or digested prior to sequencing. In preparation for Illumina sequencing, all DNA samples were sonicated in 50 µl screw cap tubes (microtube‐50, Covaris Inc., Woburn, MA, USA) using the Covaris M220 Focused Ultrasonicator (Covaris, Inc.). Specific settings achieved an average fragment size of 300 bp (Duty cycle: 10%, Intensity: 4.5, Cycles per burst: 200, Time: 120 sec), as assessed using the Agilent High Sensitivity DNA 1000 Kit on an Agilent 2100 Bioanalyzer (Agilent Biotechnologies, Santa Clara, CA, USA). Fragmented samples were prepared for sequencing using the NEBNEXT^®^ Ulta™ II DNA Library Prep Kit (New England Biolabsfor the Illumina MiSeq platform with paired end reads (2 × 150 bp). After library construction, DNA size and concentration were assessed again using an Agilent 2100 Bioanalyzer (Agilent Biotechnologies, Inc.).

We utilized the BBMap tool suite to confirm read identity and investigate contaminating non‐*P. falciparum* reads (version 38.33, https://sourceforge.net/projects/bbmap/). First, BBduk was used to remove adapters and low‐quality reads. The resulting reads were submitted to the NCBI database to identify contaminating sequences, which included human and three bacterial species. The resulting reads were then aligned to remove contamination, using the BBMap alignment algorithm, to the top four contaminating sources (human hg19 reference genome, *Bradyrhizobium* sp. BF49 genome assembly (Genbank accession GCA_900011245.1), *Escherichia*_sp_1_1_43_V2 assembly (Genbank accession GCA_000159895.2), and the *Agrobacterium* genomosp. 1 str TT111 reference genome (Genbank accession GCA_900012575.1)), represented in Table S3. The parameters used for BBMap alignment included a minimum of 95% identity, max indels of 3, a minimum of two seed hits, bwr = 0.16, and quick match and fast modes enabled. Unmapped reads from this step were presumed to be “clean” *P. falciparum* reads and were used for subsequent CNV identification (see below). These resulting reads were also used to visually assess *dhodh* amplicon properties and to identify highly enriched chromosomal locations using Geneious (Geneious Prime 2019) and Integrative Genomics Viewer (IGV 2.4.10) Software (Table S5).

For copy number determination (CN values presented in Table 3 and Table S4), we adapted the Speedseq pipeline, which incorporates BWA‐MEM alignment and automatic split/discordant read pair extraction with two CNV identification algorithms, CNVnator, and LUMPY (Chiang et al., 2015; Huckaby et al., 2019). Default settings were used for BWA‐MEM alignment of reads without adapters to the WT1 (*Dd2*) genome (PlasmoDB release 20190829). The percent of reads that mapped to reference genome, Q score, average read length, and coverage were determined using samtools (Li et al., 2009). These statistics were used to evaluate reads and the alignment quality (Table S3). Coverage across regions of interest (amplicons, chromosomes, etc) were determined using the bedtools coverage command (Quinlan and Hall, 2010). The split and discordant reads from this alignment were subsequently analyzed using LUMPY to determine the location and length of the *dhodh* amplicon (see Table S4). QualiMap 2 was also used to quantify the number of reads that aligned to the *Plasmodium* genome (see Figure S7). Lastly, to determine the orientation of the amplicon (tandem or reverse tandem duplication), discordant reads were visually inspected at the breakpoints using IGV 2.4.10 (Figure S8).

## CONFLICT OF INTEREST

There are no conflict of interest.

## AUTHOR CONTRIBUTIONS

Conceived and designed the experiments: JMM, SAC, PKR, and JLG. Performed the experiments: JMM, ACH, SAC, SLL, MC, AF, and JLG. Analyzed the data: JMM, ACH, SAC, MC, and JLG. Contributed reagents/materials/analysis tools: WS, PKR, and JLG. Wrote and edited the paper: JMM and JLG.

## Supporting information

Table S1‐S5‐Fig S1‐S8

## Data Availability

The data that support the findings of this study are available from the corresponding author upon reasonable request. The raw sequence files generated and analyzed during the current study are available in the Sequence Read Archive (SRA) under the BioProject ID PRJNA648140.
