# Supplementary material for: Extrachromosomal DNA amplicons in antimalarial‐resistant Plasmodium falciparum
Source: Mol Microbiol. 2020 Nov 19;115(4):574–90. doi: 10.1111/mmi.14624 (PMC8246734; doi:10.1111/mmi.14624)
Supplement: Supplementary file 1 — Table S1‐S5‐Fig S1‐S8 [file MMI-115-574-s001.docx]

**Title: Extra-chromosomal DNA amplicons in antimalarial resistant *Plasmodium falciparum***

Authors: Jennifer M. McDaniels^1^, Adam C. Huckaby^1^, Sabrina A. Carter^1^, Sabrina Lingeman^1^, Audrey Francis^1^, Molly Congdon^2^, Webster Santos^2^, Pradipsinh K. Rathod^3^, and Jennifer L. Guler^1, 4,^ *

**Supplemental Information**

**Supplemental Table 1. Comparison of expected and observed band sizes from restriction digestion and Southern blot analysis.**

| Probe | Category | Clone  (# of amplicons) | Expected band size (kb) | | Observed band size (approximate) (kb) | |
| --- | --- | --- | --- | --- | --- | --- |
|  |  |  | B | B+N | B | B+N |
| DHODH Amplicon | No CNV | WT1 | 79.2 | 55.4 | 80 | 50 |
|  | L1 clone derived | L1 (4) | 298* | 73.4/55.4 | 200 | 1. <80 2. 50** |
|  |  | H1 (10) | 736* | 73.4/55.4 | 250 | 1. <80 2. 50** |
|  |  | H2 (12) | 882* | 73.4/55.4 | 250 | 1. <80 2. 50** |
|  | L2 clone derived^ | L2 (4) | 79.2 | 55.4 | 80 | 50 |
|  |  | H4 (12) | 79.2 | 55.4 | 80 | 50 |
| Chr. 6 single copy gene | No CNV | WT1 | 79.2 | 55.4 | 80 | 50 |
|  | L1 clone derived | L1 (4) | 298* | 55.4 | ND | ND |
|  |  | H1 (10) | 736* | 55.4 | 250 | 50 |
|  |  | H2 (12) | 882* | 55.4 | 250 | 50 |
|  | L2 clone derived^ | L2 (4) | 79.2 | 55.4 | 80 | 50 |
|  |  | H4 (12) | 79.2 | 55.4 | 80 | 50 |

B, BamHI restriction enzyme; N, NheI restriction enzyme; ND, not determined.

*This value is dependent on the number of tandem amplicons present in the genome (due to a lack of BamHI site within the amplicon). Those listed assume 4 (L1) and 10 or 12 (H1 and H2 clones) copies of the *dhodh* amplicon, respectively (determined by qPCR of genomic DNA in Guler et al., 2013).

**This band is only present once in genome despite amplicon, so band intensity is lower than band 1.

^BamHI sites and chromosomal 6 single copy gene probe sites are within the amplicon so the full length of tandem amplicons cannot be determined in these parasite clones.

**Supplemental Table 2. Impact of exonuclease treatment on gel-incompetent DNA samples that were amplified prior to sequencing.**

| Sample ID | Treatment | %AT | Reads mapping to *P. falciparum* |
| --- | --- | --- | --- |
| H1 gel-incompetent DNA | -PS/+WGA | 80.9 | 1204943 |
|  | **+PS**/+WGA | **54.7** | **310** |
| WT1 gel-incompetent DNA | -PS/+WGA | 80.5 | 313456 |
|  | **+PS**/+WGA | **49.8** | **972** |

PS, Plasmid-Safe exonuclease; WGA, whole genome amplification. Sequencing reads were mapped to the WT1 (Dd2) reference genome (PlasmoDB release 20190829) using the BBMap alignment algorithm (version 38.33).

**Supplemental Table 3. Summary of Illumina sequencing results.**

| Samples | MapQ score  (Mean) | Insert size (median bp) | Total reads (after basic QC) | Reads mapping to human genome | Reads mapping to bacterial genomes | Reads mapping to *P. falciparum* | % of genome covered by >1 read |
| --- | --- | --- | --- | --- | --- | --- | --- |
| H1 genomic DNA | 57.6 | 224 | 1,886,432 | 323,978 | 0 | 1,542,854 | 96.3 |
| H1 gel-incompetent DNA^‡^ | 57.5 | 205 | 1,494,188 | 15,376 | 5,408 | 1,204,943 | 93.8 |
| WT1 gel-incompetent DNA^‡^ | 57.4 | 265 | 512,244 | 1,698 | 648 | 313,456 | 71.2 |

Sequencing reads were mapped to the WT1 (Dd2) reference genome (PlasmoDB release 20190829) using the BBMap alignment algorithm (version 38.33). ^‡^These samples were isolated from the PFGE well and amplified using multiple displacement amplification in order to generate enough material for sequencing. % genome covered by >1 read was determined using Qualimap 2.2.1.

**Supplemental Table 4. Copy number assessment at the super-peak on chromosome 6.**

| Samples | Chromosome 6 Coverage* | | |
| --- | --- | --- | --- |
|  | Without *dhodh* amplicon | Super-peak only^ | Estimated CN |
| H1 genomic DNA | 8.8x | 42.2x | 5^§^ |
| H1 gel-incompetent DNA^†^ | 5.8x | 31,208.2x | 5,381 |
| WT1 gel-incompetent  DNA^†^ | 1.5x | 3.6x | 2 |

*Sequencing reads were aligned to the WT1 (Dd2) reference genome using BWA-mem and chromosome (chr) 6 coverage is calculated as the mean across the chromosome, excluding amplified regions.

^†^These samples were isolated from the loading well of a PFGE gel and amplified using a whole genome amplification kit to generate enough material for sequencing.

^The super-peak is a AT-rich 714bp region that covers an intergenic region and as well as part of a genic region corresponding to the *sac3 domain-containing, putative protein* (PF3D7_0602600). ^§^This region is underrepresented compared to the CN of 9 for the *dhodh* amplicon in H1 genomic DNA (**Table 3**).

CN, relative copy number (calculated by dividing mean coverage of the amplicon by the coverage of the remainder of the chromosome); *dhodh*, dihydroorotate dehydrogenase.

**Supplemental Table 5. Summary of highly enriched chromosomal locations from H1 gel-incompetent DNA sample**

| Chromosome | Location | Size (bp) | Max Coverage | Mean Coverage |
| --- | --- | --- | --- | --- |
| 1 | 32,130-37,106 | 4,985 | 151 | 150.4 |
|  | 99,965-100,377 | 413 | 150 | 150 |
|  | 241,037-242,089 | 1,054 | 151 | 150.7 |
|  | 418,189-418,437 | 249 | 151 | 150.8 |
| 6 | **86,429-87,143** | **714** | **36,636** | **25,641** |
|  | 788,760-789,239 | 482 | 232 | 174.2 |
| 9 | 2,794-6,432 | 3,653 | 151 | 146.7 |
|  | 166,841-171,721 | 4,906 | 151 | 142.8 |
|  | 244,334-244,610 | 297 | 118 | 61.6 |
|  | 556,692-557,096 | 415 | 72 | 48.2 |
|  | 2,794-6,432 | 3,653 | 151 | 146.7 |
|  | 166,841-171,721 | 4,906 | 151 | 142.8 |
| 10 | 192,663-194,034 | 1,374 | 151 | 151 |
|  | 345,325-345,715 | 508 | 1499 | 1048.9 |
| 11 | 1,489,486-1,489,770 | 286 | 127 | 81.3 |
| 12 | 589,733-590,245 | 557 | 131 | 74.7 |
|  | 1,047,359-1,047,727 | 432 | 2013 | 1238 |
|  | 1284219-1,284,726 | 526 | 115 | 76.9 |
| 13 | 1,643,674-1,644,457 | 802 | 157 | 122.5 |
|  | 2,293,426-2,293,693 | 268 | 243 | 143.5 |
| 14 | 426,901-427,229 | 334 | 665 | 377.8 |
| Mean (minus super-peak) | | 1,505 | 337 | 239 |

The super-peak is bolded. Highly enriched chromosomal locations are defined as regions with coverage levels that are well above the mean coverage of the genome (5.5X for this sample) and were identified using Geneious (Geneious Prime 2019).

**
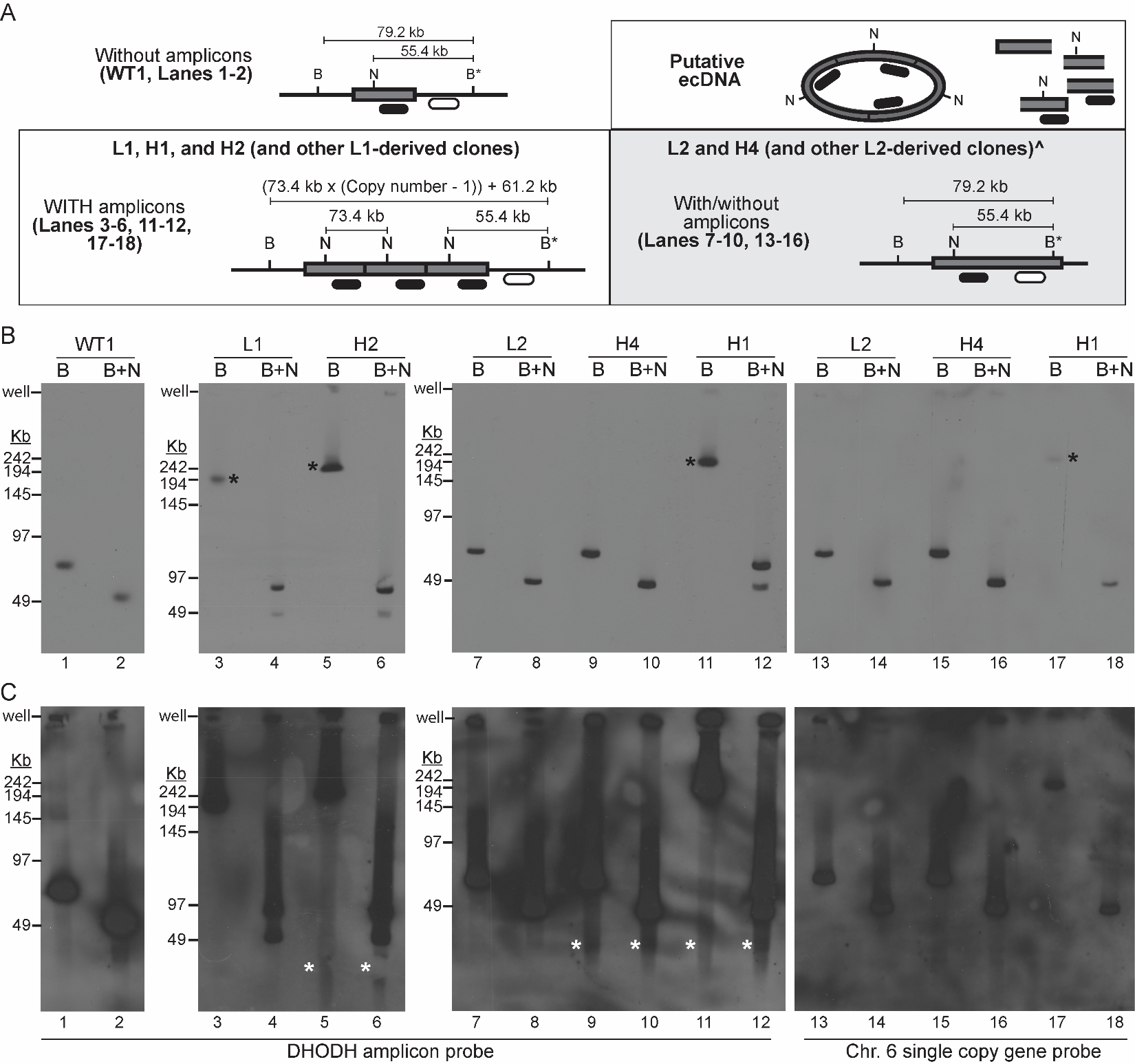
**

**Supplemental Figure 1. Southern blot analysis following restriction digestion demonstrates few chromosomal amplicons and persistence of gel-competent ecDNA. A.** Schematic of digestion pattern in genomes with and without *dhodh* amplicons. Putative ecDNA is depicted as multiple forms based on studies presented in the manuscript; not all ecDNA elements are expected to contain an amplicon probe site or restriction site. Expected and observed band sizes for all clones analyzed are displayed in **Supplemental Table 1**. All restriction sites were predicted from the 3D7 reference genome but confirmed in sequencing reads from WT1 and L clones. For L1 and derived clones (H1 and H2), the dhodh amplicon is ~70kb and only contains a single NheI site and *dhodh* probe location. For L2 and derived clones (H4), the dhodh amplicon is ~100kb and all probes and cut sites are contained within this larger amplicon. B, BamHI; N, NheI; B+N, double restriction digest; grey square, *dhodh* amplicon; black ellipse, *dhodh* probe; white ellipse, chromosomal 6 single copy gene probe located outside the *dhodh* amplicon; B*, due to varying lengths of the dhodh amplicon (see above, Guler et al., 2013), the B* site is located within L2 and H4 amplicons; **B and C.** Two exposures of a Southern blot hybridized with either the *dhodh* amplicon probe (**Table 1**, lanes 1-12) or the chromosomal 6 single copy gene probe, which sits downstream of the *dhodh* amplicon (**Table 1**, lanes 13-18). PFGE running conditions: ~50hr, 3V/cm, 250-900sec switch rate; DNA source: parasite agarose plugs; White asterisks, gel-competent ecDNA that persists following restriction digestion; WT1, Dd2. As much of the plot as possible is shown, including the well; no other hybridization signal was detected on the deleted regions. DNA size was determined with a 0.05-1Mb marker (Bio-Rad 170-3635). **B**. Exposure times: Lanes 1-12, 15-30min; Lanes 13-18, 3hr. **C**. Exposure time: Lanes 1-2 and 7-12, 15hr; Lanes 3-6, 18hr; Lanes 13-18, 17hr.


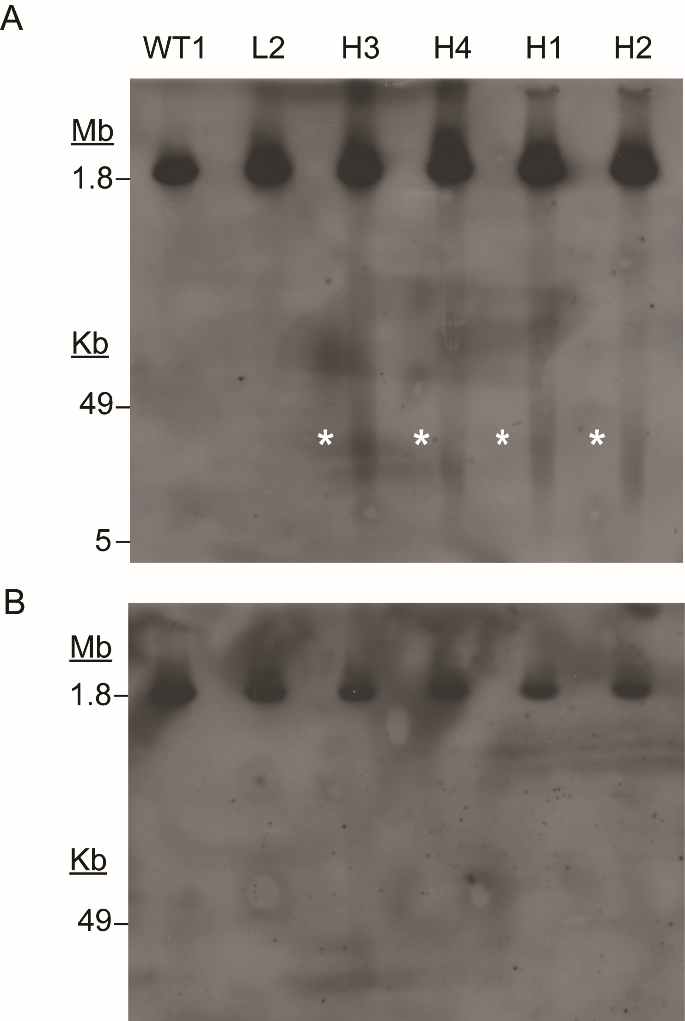


**Supplemental Figure 2. Southern blot analysis shows that additional DSM1 resistant clones all harbor ecDNA containing the *dhodh gene.*** DNA source: parasite agarose plugs made from wild type (WT1), low level resistant clone (L2), and high level resistant clones (H1-H4, Figure 1). PFGE running conditions: 17hr, 6V/cm, 1-10sec switch rate. WT1, Dd2. **A.** Southern blot hybridized with *dhodh* amplicon probe (**Table 1**). Exposure time: 11.5hr. White asterisks, gel competent ecDNA. The expected size of WT1 chromosome 6 is 1.4Mb (PlasmoDB (Aurrecoechea et al., 2009)). **B**. Southern blot hybridized with single copy reference gene 2 (Table 1). Exposure time: 16.5hr. The expected size of WT1 chromosome 7 is 1.4Mb (PlasmoDB (Aurrecoechea et al., 2009)). DNA size was determined with 0.05-1Mb and 5-120kb markers (BioRad 170- 3635 and 170-3624).

**Supplemental Figure 3. Identification of amplicon specific slow-migrating material using an alternative agarose type during PFGE.** Only the top portion of the gel including the well and larger chromosomes are displayed. DNA source: parasite agarose plugs made from wild type (WT1) and high level resistant clones (H1-H4 clones). Type of agarose used for all panels: 1% megabase agarose instead of the standard pulse field certified agarose. PFGE running conditions: 24hr, 6V/cm, 60-90sec switch rate. WT1, Dd2. **A**. PFGE gel stained with 1µg/mL ethidium bromide. Chromosomes 6-10 co-migrate at these specific electrophoresis parameters. **B.** Southern blot hybridized with *dhodh* amplicon probe (**Table 1**). Exposure time: 6hr. White asterisks, putative slow-migrating extra-chromosomal amplicons restricted to resistant parasites (H3, H4, H1, and H2). **C**. Southern blot hybridized with single copy reference gene 2 (**Table 1**). Exposure time: 16hr.

**
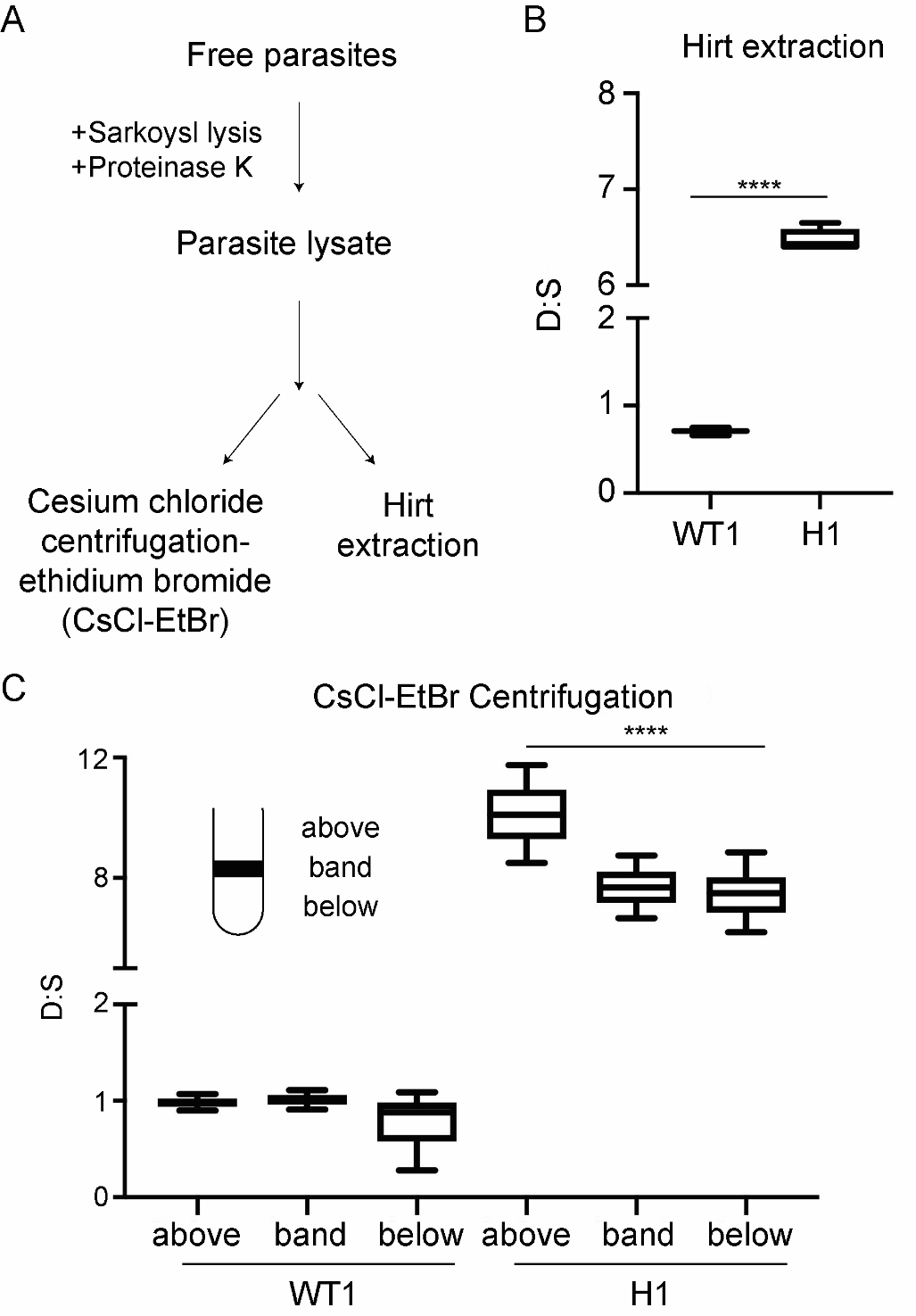
**

**Supplemental Figure 4. Alternative methods for the purification of ecDNA in highly resistant DSM1-resistant parasites.** *Note: These methods were considered unsuccessful at enriching DNA because we did not detect enrichment levels beyond what is those from genomic DNA samples.* **A.** Experimental scheme for the isolation of ecDNA. Parasites were isolated from red blood cells and then treated with a detergent and Proteinase K to release DNA in a 24hr incubation. Parasite lysate was subjected to Hirt Extraction, when DNA is purified using sodium dodecyl sulfate (SDS) and high concentration of salts, or CsCl centrifugation, when DNA is purified using isopycnic separation. **B** and **C**. Box and whisker plots depicting D:S ratio (*dhodh*: *seryl tRNA synthetase*) measured using ddPCR. Both isolation methods produced reproducible results that matched chromosomal estimates (Guler et al. 2013). N=6. **C.** Cesium chloride-ethidium bromide centrifugation of WT1 and H1 parasites produced a single visible band. The below band, band, and above band fractions (inset) were isolated, purified, and measured. WT1, Dd2; H1, high level resistant clone; ddPCR, droplet digital PCR. Error bars represent Poisson confidence intervals (upper hinge, 75th percentile; lower hinge, 25th percentile).****, p<0.0001.


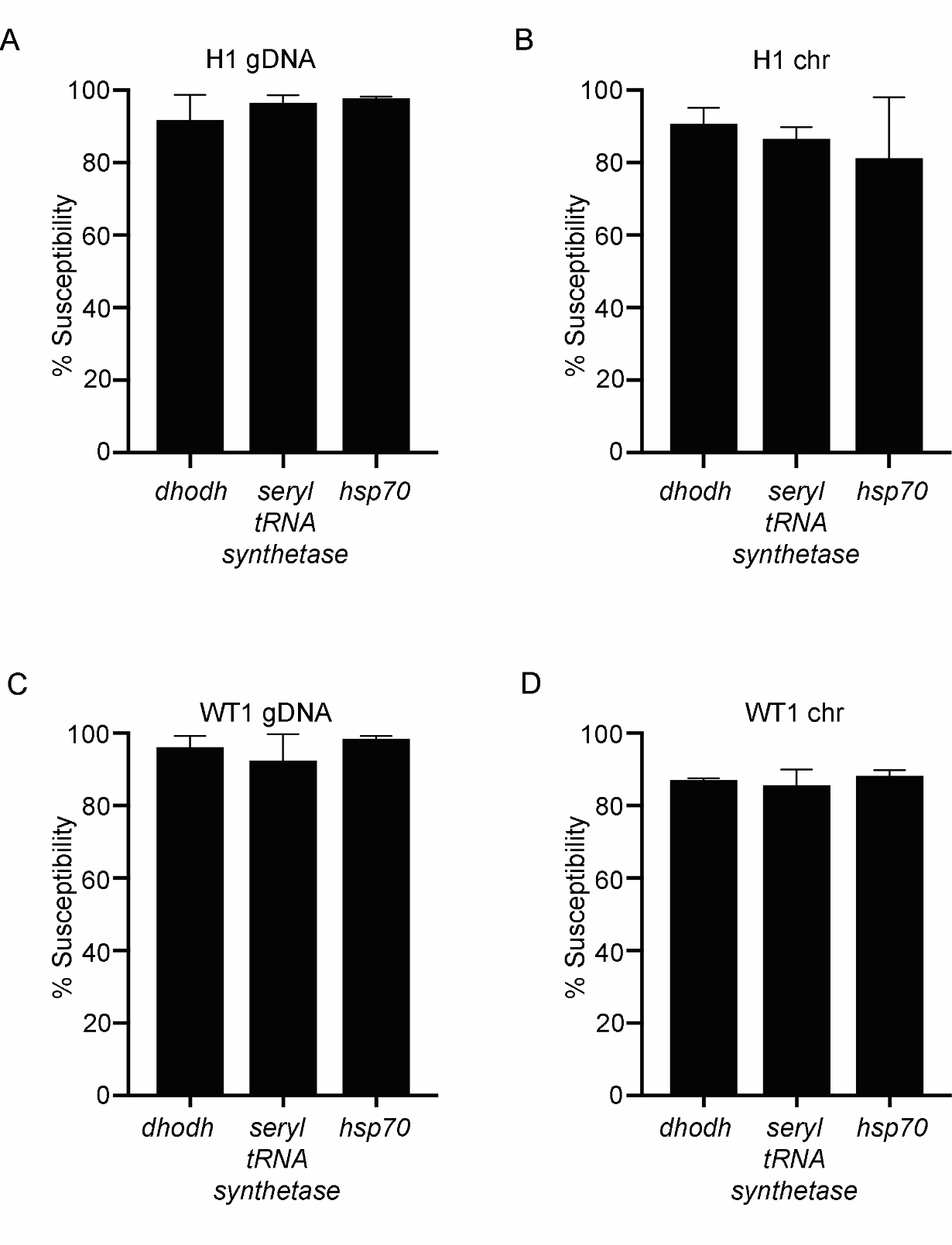


**Supplemental Figure 5. Plasmid Safe (PS) exonuclease does not exhibit loci preference.** No significant differences after PS digestion were observed at 3 different loci across the parasite genome. DNA samples were either extracted directly from red blood cells as genomic DNA (gDNA, **A and C**) or extracted from the chromosomal region of PFGE agarose gels (**B** and **D**) prior to digestion with PS and assessed by Droplet Digital (dd)PCR for specific genomic loci (*dhodh*, *dihydroorotate dehydrogenase*, chromosome 6; *seryl tRNA synthetase*, chromosome 7, and *hsp70,* chromosome 11, **Table 1**). The PS susceptibility of each genomic locus was determined by sensitively quantifying DNA-containing droplets using ddPCR; the % susceptibility (or level of DNA degradation) was calculated by dividing the number of loci-positive droplets after digestion by the number of loci-positive droplets before digestion and multiplying by 100. gDNA, genomic DNA; chr, chromosome; WT1, Dd2; H1, high level resistant clone; Error bars denote standard error, N=3.


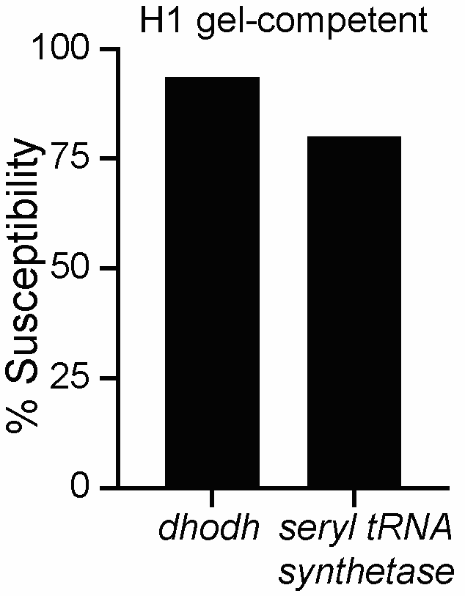


**Supplemental Figure 6. Plasmid Safe (PS) exonuclease degrades gel-competent DNA.** DNA samples were extracted from the smear region of PFGE agarose gels prior to digestion with PS and assessed by Droplet Digital (dd)PCR for specific genomic loci (*dhodh*, *dihydroorotate dehydrogenase*, chromosome 6; *seryl tRNA synthetase*, chromosome 7, **Table 1**). The PS susceptibility of each genomic locus was determined by sensitively quantifying DNA-containing droplets using ddPCR; the % susceptibility (or level of DNA degradation) was calculated by dividing the number of loci-positive droplets after digestion by the number of loci-positive droplets before digestion and multiplying by 100. H1, high level resistant clone. N=1 due to limitations in material; our purifications from the PFGE gel in this region yield very low amounts of DNA (sub-ng levels).
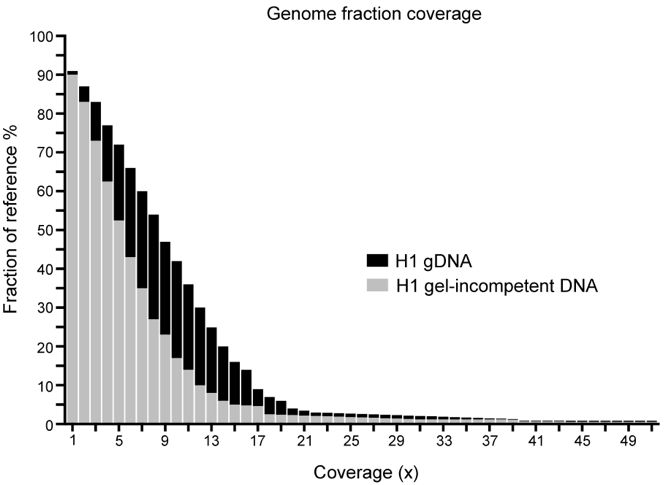


**Supplemental Figure 7. Histogram of sequencing coverage of H1 genomic (g)DNA and gel-incompetent DNA compared to the reference genome.** Plot was created using Qualimap 2.2.1. H1, high level resistant clone; gDNA, genomic DNA.


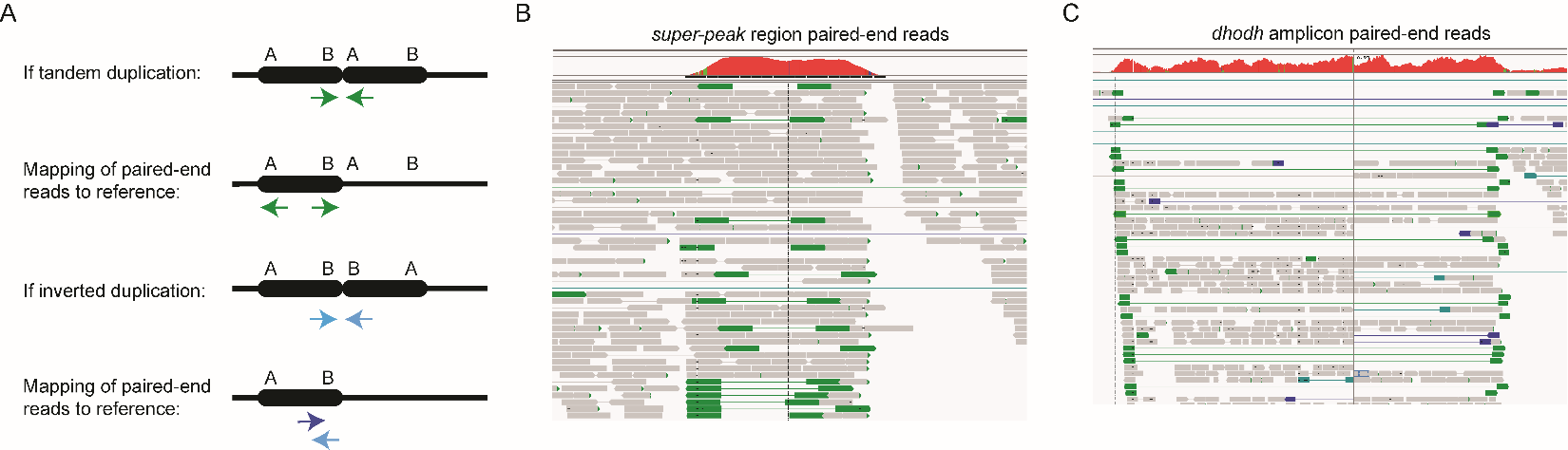


**Supplemental Figure 8. Orientation of discordant reads at *sac3* super-peak position and *dhodh* amplicon is indicative of tandem duplication. A**. Schematic of a tandem duplication, which illustrates paired-end reads pointing outwards (green arrows) when mapped to the reference genome and an inverted duplication, which illustrates paired-end reads facing each other and overlapping (blue/teal reads) when mapped to the reference genome. In this context, blue versus teal arrows depict different alignment directions. **B and C*.*** The location of discordant reads at specified locations on chromosome 6. Reads were analyzed using Integrative Genomics Viewer Software (IGV 2.4.10) and images do not depict the total reads at either location. As in panel A, colored arrows depict discordant reads: green arrows provide evidence of tandem duplications and blue/teal arrows provide evidence of inverted duplications. **B**. The location of the super-peak includes part of the gene for the *sac3 domain-containing, putative protein* found at position 86,429 - 87,143bp. H1 gel-incompetent DNA was isolated, amplified, sequenced, and the paired ends are aligned to the WT1 (Dd2) reference genome. **C**. The *dhodh* amplicon was previously reported as a tandem duplication (Guler et al., 2013). Due to the large size of the amplicon (>70kb), boundaries of reads are shown in a split screen.
